# Antioxidants into Nopal (*Opuntia ficus-indica*), Important Inhibitors of Free Radicals’ Formation

**DOI:** 10.3390/antiox10122006

**Published:** 2021-12-16

**Authors:** Romina Castañeda-Arriaga, Adriana Perez-Gonzalez, Tiziana Marino, Nino Russo, Annia Galano

**Affiliations:** 1 Departamento de Química, Universidad Autónoma Metropolitana-Iztapalapa, San Rafael Atlixco 186, Col. Vicentina, Iztapalapa, Ciudad de Mexico 09340, Mexico; animor_ca@hotmail.com (R.C.-A.); annia.galano@gmail.com (A.G.); 2 CONACYT-Universidad Autónoma Metropolitana, Iztapalapa, San Rafael Atlixco 186, Col. Vicentina, Iztapalapa, Ciudad de Mexico 09340, Mexico; adriana_perez_3@hotmail.com; 3 Dipartimento di Chimica e Tecnologie Chimiche, Universitaà della Calabria, I-87036 Arcavacata di Rende, Italy; nino.russo@unical.it

**Keywords:** natural antioxidants, fenton reactions, phenolic acids in nopal, oxidative stress, DFT

## Abstract

Nopal (*Opuntia ficus indica*) belonging to the Cactacea family has many nutritional benefits attributed to a wide variety of phenolic and flavonoid compounds. Coumaric acid (COA), ferulic acid (FLA), protocatechuic acid (PRA), and gallic acid (GAA) are the phenolic acids (PhAs) present in nopal. In this study, the role of these PhAs in copper-induced oxidative stress was investigated using the density functional theory (DFT). The PhAs form 5 thermodynamically favorable complexes with Cu(II), their conditional Gibbs free energies of reaction (ΔG’, at pH = 7.4, in kcal/mol) are from −23 kcal/mol to −18 kcal/mol. All of them are bi-dentate complexes. The complexes of PRA and GAA are capable of inhibiting the Cu(II) reduction by both O_2_^•−^ and Asc^−^, their reactions with the chelated metal are endergonic having rate constants about ~10^−5^–10^2^ M^−1^ s^−1^, PhAs can prevent the formation of hydroxyl free radicals by chelating the copper ions. Once the hydroxyl radicals are formed by Fenton reactions, the complexes of PhAs with Cu(II) can immediately react with them, thus inhibiting the damage that they can cause to molecules of biological interest. The reactions between PhAs-Cu(II) complexes and hydroxyl free radical were estimated to be diffusion-limited (~10^8^ M^−1^s^−1^). Thus, these chelates can reduce the harmful effects caused by the most reactive free radical existent immediately after it is formed by Fenton reactions.

## 1. Introduction

Several diseases are caused by oxidative stress (OS) such as cancer, rheumatoid arthritis, pulmonary and renal failures, cardiovascular diseases and neurodegenerative disorders such as Alzheimer’s and Parkinson’s diseases, multiple sclerosis, and memory loss [1,2,3,4,5,6,7,8,9,10]. OS is caused by an excess in the production of reactive oxygen species (ROS) and the inability of the body to remove such excess. The imbalance between production and consumption of ROS can severely damage cells or molecules of biological interest such as proteins or DNA [11,12]. Therefore, to preserve human health, it is important to find efficient strategies to attenuate the OS-induced molecular damage. In addition to enzymatic defense systems, chemical antioxidants arising from plant extracts containing phenolic and flavonoid compounds are a viable tool to achieve that purpose.

Nopal (*Opuntia ficus indica*) is part of the Cactacea family. This plant grows wildly in arid and semi-arid regions in America and Europe. The tender young part of the cactus stem, or cladode, is frequently consumed as a vegetable in salads, while the nopal fruit is consumed as a fresh fruit. The fruit and cladodes of nopal have nutritional benefits attributed to their antioxidant properties and the pharmacological activity of their compounds [13,14,15]. Consumption of nopal has been recommended for the treatment of some diseases like diabetes mellitus, blood glucose levels, hyperlipidemia, obesity and gastrointestinal disorders, and neuronal injury [16,17,18,19] Nopal is a valuable source of nutrients [20] as well as antiulcerogenic [21,22], antioxidant [19,20,23,24,25] anticancer [23], neuroprotective [26], hepatoprotective [27], and antiproliferative [28] compounds.

**Figure 1:**
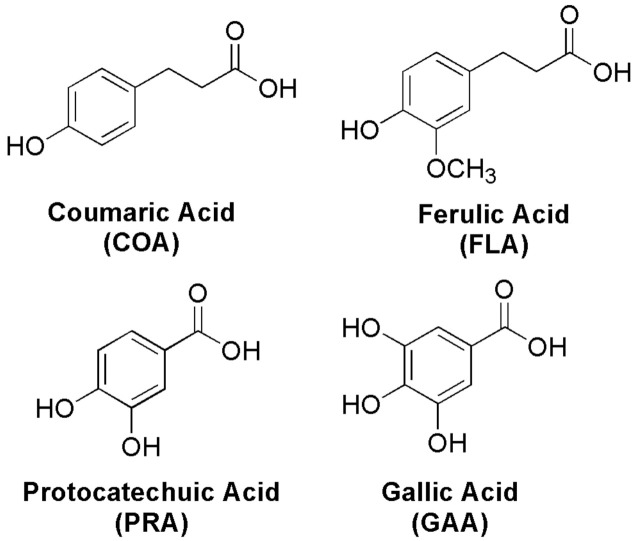
Phenolic acids (PhAs) in Nopal.

All parts of the nopal are rich in polyphenolic compounds and phenolic acids (PhAs), as is reported in Figure S1. These compounds are abundant within the plant’s kingdom and human diet. Coumaric acid (COA), ferulic acid (FLA), protocatechuic acid (PRA), and gallic acid (GAA) are PhAs present in nopal [29] (Figure 1). These PhAs, in turn, have numerous beneficial effects on human health including antibacterial [30], anticoagulatory [31], anti-inflammatory [29,32], antihyperglycemic [33], antimutagenic [34,35], antiviral [36,37], cardioprotective [38] and neuroprotective [39] activities.

Chemical composition depends on the cactus variety, maturation stage and environmental conditions of the place of origin [40,41]. Different chemical compounds have been found in the nopal, which are distributed in different proportions in its tissues. Cactus fruit contains important amounts of ascorbic acid, vitamin E, carotenoids, phenols, flavonoids, betaxanthin, betacyanin, and amino acids [42,43], while, in its flowers, many flavonoids are present, such as kaempferol and quercetin [44]. Cactus peel and seeds are rich in palmitic acid, oleic acid, and linoleic acid [45]. The cactus cladodes contain vitamins, various flavonoids, particularly quercetin 3-methyl ether, narcissin, gallic acid, and coumaric acid [46,47,48].

The growing interest in phenolic compounds results from their antioxidant potential. For example, COA, FLA, PRA and GAA have shown to have antioxidant activity as peroxyl radicals scavengers [49,50,51,52,53,54,55]. They react with the hydroperoxyl radical (^•^OOH) at rate constants of 10^5^–10^8^ M^–1^ s^–1^ in aqueous solution and at physiological pH, which indicates that, as free radical scavengers, they are better than Trolox, which is a reference compound [56,57,58].

In addition to its free radical scavenging activity, some phenolic compounds can prevent the formation of OS in the presence of redox metals such as iron or copper. These metals participate in Fenton-type reactions generating hydroxyl radicals (^•^OH) that are a highly oxidizing species. However, for COA, FLA, PRA and GAA there are not enough data to determine if they could be implicated in preventing the production of ^•^OH radicals. So, the aim of the present investigation was to gain a deeper knowledge on this activity and to provide a qualitative and quantitative analysis of the working mechanisms of COA, FLA, PRA and GAA.

## 2. Methodology

In the present investigation, the Gaussian 09 package of programs [59] was used. The computational details to study the mechanisms for reactions between the complexes and the hydroxyl free radical are in line with the quantum mechanics-based test for the overall free radical scavenging activity (QM-ORSA) protocol [60]. In the present investigation, all the calculations were carried out inside the framework of the density functional theory (DFT) and, in particular, a Thrular functional M05 [61] was used in conjunction with the basis set of Pople 6-311+G(d,p). To simulate the aqueous environment, the continuum solvent method based on density SMD [62] was considered. SMD can be safely used for estimating solvation free energies for any charged or uncharged solutes, with relatively low errors [59]. The local minima and transition states were identified by 0 and 1 imaginary frequencies, respectively. To verify that the imaginary frequency of each transition state corresponds to the expected motion along the reaction path, the intrinsic reaction coordinate (IRC) calculations were carried out. Unrestricted calculations were used for the open-shell systems. The energetic values include thermodynamic corrections at 298.15 K.

The conventional transition state theory (TST) [63,64,65] and the 1 M standard state were used to calculate the rate constants, using harmonic vibrational frequencies and Eckart tunneling [66]. For the electron transfer reactions, the Gibbs free energy of activation were calculated using the Marcus theory [67]. In addition, since several of the calculated rate constants (*k*) are close to the diffusion limit, the apparent rate constant (*k_app_*) cannot be directly obtained from TST calculations. The Collins–Kimball theory is used to that purpose [68], in conjunction with the steady-state Smoluchowski [69] rate constant for an irreversible bimolecular diffusion-controlled reaction, and the Stokes–Einstein [70] approaches for the diffusion coefficient of the reactants.

In the complexes, the Cu (II) ions were modeled coordinated to water molecules, because they are expected to be hydrated in the aqueous phase. This model results to be more adequate to represent ‘‘free’’ copper in biological systems than the corresponding naked ions. Four water molecules were chosen for this purpose, since it was previously reported that the most likely configuration of Cu (II) water complexes, in the aqueous phase, corresponds to an almost square planar four-coordinate geometry [71].

## 3. Results and Discussion

To determine the molar fractions ^M^*f* of each acid-base species, at physiological pH (pH = 7.4), it is important to take into account the pKa values; in Table 1 are reported those for the investigated PhAs. For each PhA, in Figure S2, are the deprotonation routes.

At pH = 7.4 the species with more molar fraction ^M^*f* of the investigated PhAs is the mono-anionic species (H_n−1_A^−^), but also the neutral and di-anionic species were taken into account in the present investigation. On the other hand, the molar fractions of tri-anions (H_n−3_A^−3^) and fourth anions (H_n−4_A^−4^) for PRA and GAA are almost negligible.

### 3.1. Pro-Oxidant Effects by Cu(II) Reduction

A crucial feature for the antioxidant protection, when it takes place in the presence of metal ions, is the reductant capability of the antioxidant, in particular of their deprotonated species. Since the mono-anions of the antioxidants can behave as nucleophilic agents, they can cause the reduction of copper (II) to copper (I) and therefore accelerate the Fenton reactions, thus originating hydroxyl radicals. Such pro-oxidant effects were also explored in this work, considering both PhAs and their corresponding anions (1)–(3). To put the calculated data into perspective, the Cu(II) reductant activity of the investigated PhAs was compared to those of the superoxide radical anion (O_2_^•−^), reaction (4), and the ascorbate anion (Asc^−^), reaction (5). The superoxide anion radical (O_2_^•−^) and ascorbate (Asc^−^) are reducing species at a physiological level, even at an experimental level a mixture of copper-ascorbate is used to provoke redox conditions, and the O_2_^•−^ is the main reducing species in Fenton reactions; therefore, they are used in the present investigation to analyze the reductant effect against Cu(II).

H_n_PhAs + Cu(II) → H_n_PhAs ^•+^ + Cu(I) (1)

H_n−1_PhAs^−^ + Cu(II) → H_n−1_PhAs ^•^ + Cu(I) (2)

H_n−2_PhAs^2−^ + Cu(II) → H_n−2_PhAs ^•−^ + Cu(I) (3)

O_2_^•−^ + Cu(II) → O_2_ + Cu(I) (4)

Asc^−^ + Cu(II) → Asc ^•^ + Cu(I) (5)

In Table 2 are collected the data referring to the reduction of free Cu(II). To obtain the rate constants, the molar fractions of PhAs, O_2_^•−^ and Asc^−^ were included at the pH of interest. Taking into account that the reduction of Cu(II) to Cu(I) by O_2_^•−^ experimentally occurs at 8.1 × 10^9^ M^−1^s^−1^ [75], it can be evinced that the rate constant calculated for that reaction is found to be only 1.74 times lower than the experimentally measured one. This finding supports us on the kinetic data reported and discussed in this work.

At physiological pH, the di-anionic PhAs are predicted to reduce Cu(II) to Cu(I) at significant rates. However, neutral, and mono-anionic acids do not have this pro-oxidant effect since their rate constants are around 10^−4^ M^−1^s^−1^–10^6^ M^−1^s^−1^ and the reduction of the metal by O_2_^•−^ and Asc^−^ occurs at 10^9^ M^−1^s^−1^ and 10^8^ M^−1^s^−1^, respectively. The PhAs featuring a pro-oxidant effect consist of the di-anion of gallic acid with a rate constant for the Cu(II) reduction of 1.01 × 10^9^ M^−1^s^−1^, very closed to that of O_2_^•−^, at pH = 7.4. The rate constant for the Cu(II) reduction by di-anions of COA, FLA and PRA are similar to that involving Asc^−^.

### 3.2. ^•^OH-Inactivating Ligand Behavior

The possibility that PhAs behave as ^•^OH-inactivating ligand (OIL) [76,77,78] in the presence of redox metal ions also was explored. Such a behavior can be exhibited in two different ways [72]:

OIL−1: by sequestering metal ions from reductants.

OIL−2: by deactivating ^•^OH as they are formed through Fenton-like reactions.

In both cases, OIL molecules should act as metal chelating agents. When they behave as OIL−1 agents, the metal (Cu (II)) is protected by the antioxidant in the complex formed. Thus, initially the Fenton reactions that originate hydroxyl radicals are inhibited.

Furthermore, the antioxidant behaves like OIL−2, when once the hydroxyl radical is formed by Fenton reactions, the complex of PhAs with Cu(II) immediately reacts with the radical, thus behaving as an immediate target and thus protecting other molecules of great interest such as proteins or even DNA.

Chelation was the first aspect explored here since it represents a necessary and crucial step in both cases. Cu(II) ions were modeled coordinated to four water molecules, in a near square-planar configuration and Cu(I) with four explicit water molecules too, in a linear two-coordinate configuration. In this case, in fact, Cu(I) is coordinated only to two water molecules, while the other two are retained in the second coordination shell of the metal ion.

All the possible chelation sites involving O atoms of the examined PhAs were explored, as described in detail in Table S1. In addition, their roles as mono-dentate and bi-dentate ligands have both been considered. Six different chemical routes leading to Cu(II) chelation were investigated. They are:

Route (I): Direct chelation by neutral phenols (H_n_PhAs):

Cu(H_2_O)_4_^2+^ + H_n_PhAs ⇆ [Cu(H_2_O)_4-*j*_(H_n_PhAs)]^2+^ + *j*H_2_O

Route (II): Coupled deprotonation-chelation involving neutral phenols (H_n_PhAs):

Cu(H_2_O)_4_^2+^ + H_n_PhAs ⇆ [Cu(H_2_O)_4-*j*_(H_n−1_PhAs)]^+^ + *j*H_2_O + H^+^

Route (III): Direct chelation by anionic phenols (H_n−1_PhAs ^−^):

Cu(H_2_O)_4_^2+^ + H_n−1_PhAs ^−^ ⇆ [Cu(H_2_O)_4-*j*_(H_n−1_PhAs)]^+^ + *j*H_2_O

Route (IV): Coupled deprotonation-chelation involving anionic phenols (H_n−1_PhAs ^−^):

Cu(H_2_O)_4_^2+^ + H_n−1_PhAs ^−^ ⇆ [Cu(H_2_O)_4-*j*_(H_n−2_PhAs)] + *j*H_2_O + H^+^

Route (V): Direct chelation by di-anionic phenols (H_n−2_PhAs ^2−^):

Cu(H_2_O)_4_^2+^ + H_n−2_PhAs ^2−^ ⇆ [Cu(H_2_O)_4-*j*_(H_n−2_PhAs)] + *j*H_2_O

Route (VI): Coupled deprotonation-chelation involving di-anionic phenols (H_n−2_PhAs ^2−^):

Cu(H_2_O)_4_^2+^ + H_n−2_PhAs ^2−^ ⇆ [Cu(H_2_O)_4-*j*_(H_n−3_PhAs)]^-^ + *j*H_2_O + H^+^

Table S1 collects all the complexes formed between PhAs and Cu(II) indicating their chelation routes (ChR), chelation sites (ChS), conditional Gibbs free energies of reaction (ΔG’, at pH = 7.4, in kcal/mol), and Maxwell–Boltzmann distribution (%MB) for the different chelation pathways. The corresponding equilibrium constants and Gibbs energies of reaction would explicitly depend on the pH, because the coupled deprotonation-chelation mechanisms (CDCM), i.e., routes II, IV and VI, simultaneously involve Cu(II) chelation and deprotonation of the reactive site in the ligand. Thus, to take this effect into account, the reported data correspond to pH = 7.4. If the complex corresponds to a neutral species and mono-dentate, the label is, for example, H_n_COA(**1**)-C*i*, where “*i*” is the number of the chelate. In addition, if it is from a mono-anionic species and bi-dentate, the label is, for example, H_n−1_FLA^–^(**2**)-C*i*, and so on. Some of these complexes could be formed through two routes; for example, the complex H_n_COA(**1**)-C2 formed by the route II, and the complex H_n−1_COA^–^(**1**)-C1 formed by the route III, are both the same complex (see Table S1).

Table S1 reports the values of Cu(II) chelation, by all the PhAs present in the nopal, characterized by at least one significantly exergonic pathway. In addition, for COA, PRA and GAA only one main complex is expected, with contributions larger than 98%, i.e., H_n−2_COA^2–^ (**2**)-C2, H_n−2_PRA^2–^ (**2**)-C7 and H_n−2_GAA^2–^ (**2**)-C7. For FLA, two complexes are the most abundant, H_n−2_FLA^2–^ (**2**)-C5 and H_n−2_FLA^2–^ (**2**)-C2, with 81.61% and 18.21%, respectively (Table 3). Therefore, they were the ones that have been explored as antioxidants with the behavior of OIL−1.

To this purpose, two different reductants were considered: the superoxide radical anion (O_2_^•−^) and the ascorbate anion (Asc^−^) (6). Table 4 shows Gibbs free energies of reaction and rate coefficients for the reductant reactions of the PhAs-Cu(II) complexes with the O_2_^•−^ and Asc^−^; these are compared with the reaction of Cu(II)(H_2_O)_4_ and the same reductants.

PhAs-Cu(II) + O_2_^•−^/Asc^−^ → PhAs-Cu(I) + O_2_/Asc^•^ (6)

The complexes of protocatechuic acid and gallic acid are capable of inhibiting the Cu(II) reduction by both O_2_^•−^ and Asc^−^, then they act as highly performing OIL−1. Their reactions with the chelated metal are endergonic, having rate constants about ~10^−5^–10^2^ M^−1^ s^−1^ compared with the Cu(II)(H_2_O)_4_ + O_2_^•−^ and Cu(II)(H_2_O)_4_ + Asc^−^, which are exergonic reactions with rate constants of 4.66 × 10^9^ and 1.33 × 10^8^ M^−1^ s^−1^, respectively (Table 2). On the contrary, coumaric acid and ferulic acid are able to prevent the Cu(II) reduction from Asc^−^ but do not inhibit the reduction by O_2_^•−^, albeit the reactions with these complexes are slower than with Cu(II)(H_2_O)_4_.

These results suggest that, although COA and FLA are able to coordinate copper, it is possible that the chelated metal can be reduced by strong reductants, such as O_2_^•−^, producing ^•^OH radicals, through Fenton-type reactions. This is not expected to happen if PRA and GAA coordinate copper.

**Scheme 1:**
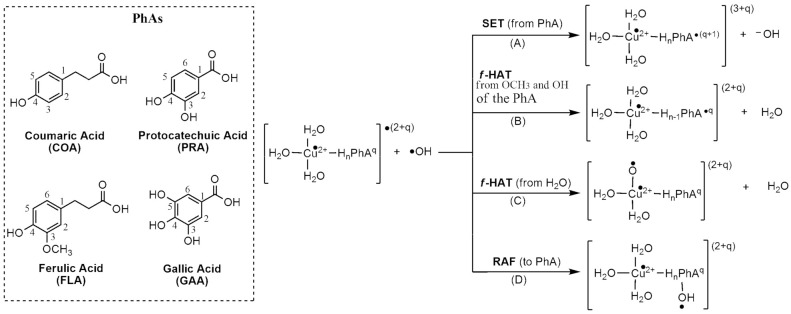
(OIL−2) of PhAs-Cu(II).

The OIL−2 behavior was also analyzed, which involves the reactions between the PhAs ligands in the copper complex and the hydroxyl radical (^•^OH). Different reaction mechanisms may contribute to such reactions, namely, single-electron transfer (SET), formal hydrogen atom transfer (*f*-HAT) and radical adduct formation (RAF) (Scheme 1). The complexes with PRA and GAA were included in this part to test if they can scavenge ^•^OH radicals formed, even though they do not contribute to their production.

Thermodynamic and kinetic results for the SET mechanism (Path (A) of Scheme 1) are collected in Table 5. The rate constants values for this mechanism are in the range of 10^–7^–10^–9^ M^–1^ s^–1^. The complex that reacts faster with ^•^OH is H_n−2_FLA^–2^-(2)-C5, while the slowest reactions correspond to that of H_n−2_FLA^–2^-(2)-C2. When copper is coordinated only with water molecules, Cu(II)(4H_2_O), the value of the Gibbs free energy of reaction with ^•^OH through the SET mechanism becomes 73.85 kcal/mol [79]. It implies that the high values in the rate constants (Table 5) are due to the presence of PhAs in the coordination sphere of Cu(II).

Thermodynamic results for *f*-HAT mechanisms (Paths (B) and (C) of Scheme 1) are reported in Table 6. All the reactions resulted to be exergonic. COA forms complexes with copper through its fully deprotonated specie. For this reason, the only hydrogens available to be transferred to ^•^OH are those of the water molecules coordinated to copper (Path (C) of Scheme 1). However, the copper complexes with PRA, FLA and GAA as ligands have protonated functional groups that also participate in *f*-HAT reactions with the ^•^OH radical (Path (B) of Scheme 1). These groups are OCH_3_ in the FLA complexes and OH in the PRA and GAA complex.

The *f*-HAT reactions are highly exergonic, with values of Gibbs free energy of reaction ranging from −41.90 to −51.76 kcal/mol, except those originated from the OCH_3_ group in the FLA complexes, the values of which are −23.63 and −21.83 kcal/mol.

The *f*-HAT reactions from water molecules binding to copper (Path (C) of Scheme 1) were identified as barrierless (Figure S3), with rate constants (k) controlled by diffusion (Table 6). The same *f*-HAT mechanism was tested for the reaction between the Cu(II)(4H_2_O) complex and the ^•^OH radical. It was found that this reaction is endergonic by 1.6 kcal/mol. Those results evidence that Cu(II) coordinated only with water molecules is not able to act as radical scavenger.

**Scheme 2:**
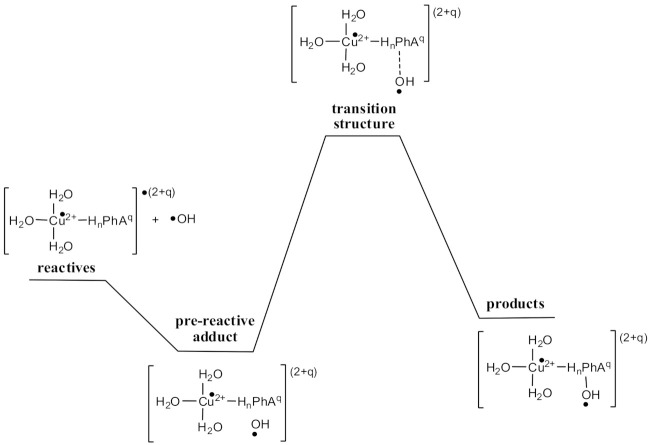
General RAF mechanism for reactions between PhAs-Cu(II) and ^•^OH.

For the RAF mechanism (Path (D) of Scheme 1), all the possible sites on the aromatic ring of PhAs numbered in Scheme 1 were considered. The Gibbs free energies of reaction (ΔG), and Gibbs free energies of activation (ΔG^≠^) through direct addition, are reported in Table S2, while the transition structures are presented in Schemes S4–S8. The ΔG energy values indicate exergonic reactions. However, the ΔG^≠^ values are negative, suggesting that, in the RAF mechanism, a more complex mechanism is involved, probably implicating the formation of a pre-reactive adduct as a crucial step before the addition reaction (Scheme 2). Therefore, in this case, the RAF reactions were evaluated considering the pre-reactive adduct and not the separated species as the RAF reactants (Table 7). From the results, it emerges that the RAF mechanism does not contribute to the OIL−2 behavior of PhAs, because all the RAF pathways are endergonic processes with high activation barriers.

## 4. Conclusions

Nopal is a plant containing four phenolic acids: coumaric acid (COA), ferulic acid (FLA), protocatechuic acid (PRA), and gallic acid (GAA). These compounds had been shown to be efficient Cu(II) chelators, yielding five complexes with high thermodynamic stability. This implies that they can be efficient in treatments to detoxify the body from copper.

Dianionic species of PhAs are capable of reducing Cu(II) to Cu(I) and therefore promote the formation of **^•^**OH radicals through Fenton-type reactions; for this reason, they can be proposed as species with a behavior of pro-oxidants. However, when the chelates are formed, they can inhibit the reduction of the metal by the reductants of the O_2_^•−^ and Asc^−^, i.e., they can be proposed to act as OIL−1, especially the chelates of PRA and GAA, whereas the chelates of COA and FLA inhibit the reduction of Cu(II) by ascorbate and only slightly the reduction by the radical anion superoxide (O_2_^•−^).

All the complexes between Cu(II) and PhAs can deactivate the ^•^OH radical through SET and *f*-HAT reactions that take place at diffusion-controlled rates. Therefore, they can act as OIL−2 agents, inhibiting the damage by hydroxyl free radical immediately as it is formed by Fenton reactions. The PhAs studied in this investigation are proposed as OIL−1 and OIL−2, and therefore they can be proposed as protectors of biomolecules since they inhibit the reduction of Cu(II) to Cu(I) and therefore the formation of the hydroperoxyl free radical, and additionally, they react with hydroxyl free radical once it is formed.

## Figures and Tables

**Table 1:** Experimental *p*K_a_ values (with their references) and the molar fractions (^M^*f*) for the different acid-base species of the investigated PhAs, at pH = 7.4.

PhAs	*p*K*_a_*_1_	*p*K*_a_*_2_	*p*K*_a_*_3_	*p*K*_a_*_4_	^M^*f* (H_n_A)	^M^*f* (H_n−1_A^−^)	^M^*f* (H_n−2_A^−2^)	^M^*f* (H_n−3_A^−3^)	^M^*f* (H_n−4_A^−4^)	Refs.
COA	4.38	8.68			0.001	0.949	0.050			[56,72,73]
FLA	4.56	8.65			0.001	0.945	0.053			[56,73]
PRA	4.38	8.74	10.67		0.001	0.955	0.044	<0.001		[56,57]
GAA	4.32	8.24	9.97	13.1	0.001	0.871	0.127	<0.001	<0.001	[58,74]

**Table 2:** Gibbs free energies of reaction (ΔG, kcal/mol), reorganization energies (λ, kcal/mol), reaction barriers (ΔG^≠^, kcal/mol) and rate constants (k, M^−1^s^−1^) for the Cu(II) reduction by PhAs, O_2_^•−^ and Asc^−^, at 298.15 K and pH = 7.4.

	∆G	λ	∆G^≠^	k
O_2_^•−^	−24.01	51.87	3.74	4.66 × 10^9^
Asc^−^	−4.67	34.14	6.36	1.33 × 10^8^
H_n_COA	12.90	29.40	15.22	4.34 × 10^−2^
H_n−1_COA^–^	5.49	30.79	10.69	8.59 × 10^4^
H_n−2_COA^2–^	−16.64	28.53	1.24	3.96 × 10^8^
H_n_FLA	8.46	29.45	12.20	7.11 × 10^0^
H_n−1_FLA^–^	2.94	29.86	9.00	1.47 × 10^6^
H_n−2_FLA^2–^	−19.39	28.45	0.72	4.22 × 10^8^
H_n_PRA	15.69	30.97	17.57	8.12 × 10^−4^
H_n−1_PRA^–^	8.14	31.35	12.43	4.57 × 10^3^
H_n−2_PRA^2–^	−16.15	29.96	1.59	3.45 × 10^8^
H_n_GAA	15.19	31.05	17.22	1.48 × 10^−3^
H_n−1_GAA^–^	8.50	31.20	12.63	2.97 × 10^3^
H_n−2_GAA^2–^	−17.80	30.11	1.26	1.01 × 10^9^

**Table 3:** Conditional Gibbs free energies of reaction (ΔG’, at pH = 7.4, in kcal/mol), and Maxwell–Boltzmann distribution (%MB) for the main complexes of PhAs and Cu(II).

Complex			ΔG’	%MB
H_n−2_COA^2–^ (2)-C2	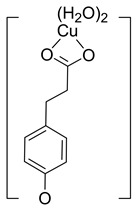	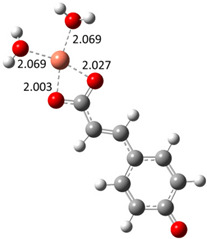	−22.87	~100
H_n−2_FLA^2–^ (2)-C5	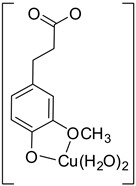	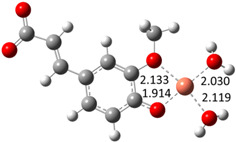	−19.19	81.61
H_n−2_FLA^2–^ (2)-C2	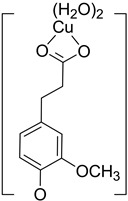	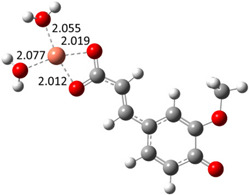	−18.30	18.21
H_n−2_PRA^2–^ (2)-C7	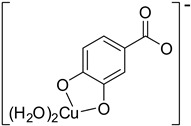	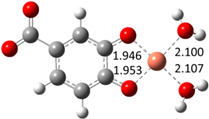	−22.83	99.99
H_n−2_GAA^2–^ (2)-C7	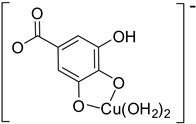	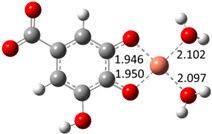	−20.83	98.05

**Table 4:** Gibbs free energies of reaction (ΔG, kcal/mol), and rate coefficients (k, M^−1^ s^−1^), for the reactions of the PhAs-Cu(II) complexes with the reductants O_2_^•−^ and Asc^−^, in aqueous solution at 298.15 K and pH = 7.4.

	O_2_^•−^		Asc^−^	
	ΔG	k	ΔG	k
CuII (H_2_O)_4_	−24.01	4.67 × 10^9^	−4.67	1.33 × 10^8^
H_n−2_COA^2–^ (2)-C2	−15.06	4.61 × 10^7^	4.28	3.19 × 10^4^
H_n−2_FLA^2–^ (2)-C5	−13.92	1.29 × 10^8^	5.41	6.62 × 10^4^
H_n−2_FLA^2–^ (2)-C2	−14.59	3.53 × 10^7^	4.74	2.09 × 10^4^
H_n−2_PRA^2–^ (2)-C7	4.13	1.39 × 10^2^	23.46	1.57 × 10^−5^
H_n−2_GAA^2–^ (2)-C7	1.35	4.75 × 10^2^	20.69	3.87 × 10^−4^

**Table 5:** Gibbs free energy of reaction (ΔG, kcal/mol), Reorganization energies (λ, kcal/mol), Gibbs free energy of activation (ΔG^≠^, kcal/mol), and rate constants (k, M^–1^ s^–1^) for the SET reactions between PhAs-Cu(II) and ^•^OH.

PhAs-Cu(II)	∆G	λ	∆G^≠^	k
H_n−2_COA^–2^-(2)-C2	−31.72	14.01	5.59	4.64 × 10^8^
H_n−2_FLA^–2^-(2)-C5	−28.01	17.36	1.63	7.36 × 10^9^
H_n−2_FLA^–2^ -(2)-C2	−35.61	14.76	7.37	2.46 × 10^7^
H_n−2_PRA^–2^-(2)-C7	−38.90	17.24	6.81	6.29 × 10^7^
H_n−2_GAA^–2^-(2)-C7	−39.36	17.41	6.92	5.21 × 10^7^

**Table 6:** Gibbs free energy of reaction (ΔG, kcal/mol) and rate constants (k, M^–1^ s^–1^) for the *f*-HAT reactions between PhAs-Cu(II) and ^•^OH.

PhAs-Cu(II)	∆G	k
H_n−2_COA^–2^-(2)-C2 from H_2_O	−42.21	2.81 × 10^9^
H_n−2_FLA^–2^-(2)-C5 from H_2_O	−41.90	8.07 × 10^9^
H_n−2_FLA^–2^-(2)-C5 from FLA (site OCH_3_)	−23.63	8.06 × 10^9^
H_n−2_FLA^–2^-(2)-C2 from H_2_O	−46.28	8.08 × 10^9^
H_n−2_FLA^–2^-(2)-C2 from FLA (site OCH_3_)	−21.83	8.08 × 10^9^
H_n−2_PRA^–2^-(2)-C7 from H_2_O	−46.80	7.96 × 10^9^
H_n−2_GAA^–2^-(2)-C7 from GAA (site OH)	−51.76	8.03 × 10^9^
H_n−2_GAA^–2^-(2)-C7 from H_2_O	−47.55	8.03 × 10^9^

**Table 7:** Gibbs free energy of reaction (ΔG, kcal/mol), Gibbs free energy of activation (ΔG^≠^, kcal/mol) and rate constants (k, M^–1^ s^–1^) for the RAF mechanism between PhAs-Cu(II) and ^•^OH, since pre-reactive adduct. Solvent = water.

PhAs-Cu(II)		ΔG	ΔG^≠^	k
H_n−2_COA^2–^-(2)-C2	RAF-1	11.18	17.53	8.76 × 10^−1^
H_n−2_COA^2–^-(2)-C2	RAF-2	6.35	17.90	4.72 × 10^−1^
H_n−2_COA^2–^-(2)-C2	RAF-3	2.19	12.46	4.57 × 10^3^
H_n−2_COA^2–^-(2)-C2	RAF-4	5.64	12.99	1.85 × 10^3^
H_n−2_FLA^2–^-(2)-C5	RAF-1	12.43	20.11	1.13 × 10^−2^
H_n−2_FLA^2–^-(2)-C5	RAF-2	NF	-	-
H_n−2_FLA^2–^-(2)-C5	RAF-3	4.18	17.81	5.44 × 10^−1^
H_n−2_FLA^2–^-(2)-C5	RAF-4	1.80	14.54	1.37 × 10^2^
H_n−2_FLA^2–^-(2)-C5	RAF-5	7.38	18.15	3.06 × 10^−1^
H_n−2_FLA^2–^-(2)-C5	RAF-6	5.07	16.21	8.17 × 10^0^
H_n−2_FLA^2–^-(2)-C2	RAF-1	13.57	18.67	1.28 × 10^−1^
H_n−2_FLA^2–^-(2)-C2	RAF-2	6.29	18.36	2.14 × 10^−1^
H_n−2_FLA^2–^-(2)-C2	RAF-3	0.84	14.43	1.63 × 10^2^
H_n−2_FLA^2–^-(2)-C2	RAF-4	5.90	11.70	1.64 × 10^4^
H_n−2_FLA^2–^-(2)-C2	RAF-5	6.72	15.12	5.09 × 10^1^
H_n−2_FLA^2–^-(2)-C2	RAF-6	5.60	15.80	1.61 × 10^1^
H_n−2_PRA^2–^-(2)-C7	RAF-1	9.83	18.93	8.24 × 10^−2^
H_n−2_PRA^2–^-(2)-C7	RAF-2	8.12	16.83	2.88 × 10^0^
H_n−2_PRA^2–^-(2)-C7	RAF-3	7.65	13.14	1.44 × 10^3^
H_n−2_PRA^2–^-(2)-C7	RAF-4	6.18	12.53	4.05 × 10^3^
H_n−2_PRA^2–^-(2)-C7	RAF-5	10.26	17.98	4.12 × 10^−1^
H_n−2_PRA^2–^-(2)-C7	RAF-6	5.52	14.53	1.38 × 10^2^
H_n−2_GAA^2–^-(2)-C7	RAF-1	11.60	20.12	1.10 × 10^−2^
H_n−2_GAA^2–^-(2)-C7	RAF-2	8.25	18.85	9.40 × 10^−2^
H_n−2_GAA^2–^-(2)-C7	RAF-3	10.66	15.74	1.79 × 10^1^
H_n−2_GAA^2–^-(2)-C7	RAF-4	6.53	13.42	9.05 × 10^2^
H_n−2_GAA^2–^-(2)-C7	RAF-5	7.30	17.40	1.09 × 10^0^
H_n−2_GAA^2–^-(2)-C7	RAF-6	8.74	17.03	2.05 × 10^0^

NF = not formed.

## Data Availability

Data is contained within the article.

## Supplementary Materials

The following are available online at https://www.mdpi.com/article/10.3390/antiox10122006/s1.
