# Supplementary material for: Antioxidants into Nopal (Opuntia ficus-indica), Important Inhibitors of Free Radicals’ Formation
Source: Antioxidants (Basel). 2021 Dec 16;10(12):2006. doi: 10.3390/antiox10122006 (PMC8698577; doi:10.3390/antiox10122006)
Supplement: Supplementary file 1 [file antioxidants-10-02006-s001.zip › antioxidants-1475301-supplementary.pdf]

Supplementary Materials

Antioxidants into Nopal (*Opuntia ficus-indica*), important inhibitors of free radicals' formation.

Romina Castañeda-Arriaga,[1] Adriana Perez-Gonzalez,[2] Tiziana Marino,[*3] Nino Russo,[3] Annia Galano,[1]

[1]*Departamento de Química. Universidad Autónoma Metropolitana-Iztapalapa. San Rafael Atlixco 186, Col. Vicentina. Iztapalapa. C. P. 09340. México D. F. México.*

[2]*CONACYT, Universidad Autónoma Metropolitana, Iztapalapa, San Rafael Atlixco 186, Col. Vicentina. Iztapalapa. C. P. 09340. México D. F. México.*

[3]*Dipartimento di Chimica e Tecnologie Chimiche, Universitàdella Calabria, I-87036 Arcavacata di Rende, Italy*

Table of Contents

Figure S1. Phenolic and poliphenolic compounds in Nopal (Opuntia ficus-indica). 3

Figure S2. Deprotonation routes of PhAs... 4

Table S1. Chelation routes (ChR), chelation sites (ChS), conditional Gibbs free energies of reaction (ΔG', at pH=7.4, in kcal/mol), and Maxwell-Boltzmann distribution (%MB) for the chelation pathways of PhAs.. 5

Figure S3. Energy scan for the *f*-HAT reactions between $^{\bullet}OH$ and PhAs-Cu(II) at 298.15 K. Solvent=water. 24

Table S2. Gibbs free energy of reaction (ΔG, kcal/mol) and Gibbs free energy of activation (ΔG$^{\neq}$, kcal/mol) for the direct RAF mechanism between PhAs-Cu(II) and $^{\bullet}OH$. Solvent=water. 25

Figure S4. Transition states structures of exergonic RAF reactions between $^{\bullet}OH$ and $H_{n-2}COA^{2-}$-(2)-C2 at 298.15 K. Solvent=water. 26

Figure S5. Transition states structures of exergonic RAF reactions between $^{\bullet}OH$ and $H_{n-2}FLA^{2-}$-(2)-C5 complexes at 298.15 K. Solvent=water. 27

Figure S6. Transition states structures of exergonic RAF reactions between $^{\bullet}OH$ and $H_{n-2}FLA^{2-}$-(2)-C2 complexes at 298.15 K. Solvent=water. 28

Figure S7. Transition states structures of exergonic RAF reactions between $^{\bullet}OH$ and $H_{n-2}PRA^{2-}$-(2)-C7 complexes at 298.15 K. Solvent=water. 29

Figure S8. Transition states structures of exergonic RAF reactions between $^{\bullet}OH$ and $H_{n-2}GAA^{2-}$-(2)-C7 complexes at 298.15 K. Solvent=water. 30

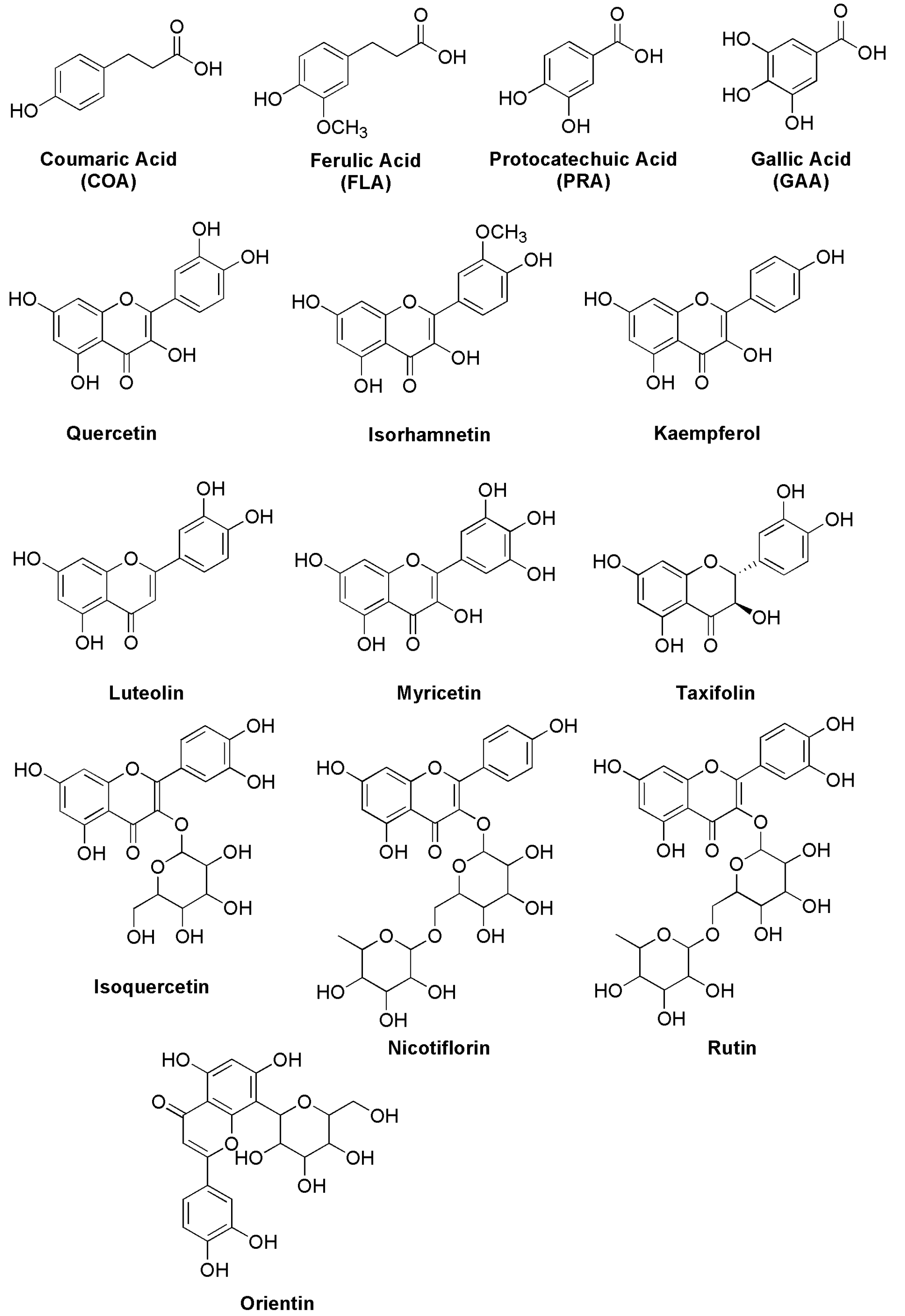

Figure S1. Phenolic and polyphenolic compounds in Nopal (*Opuntia ficus-indica*).

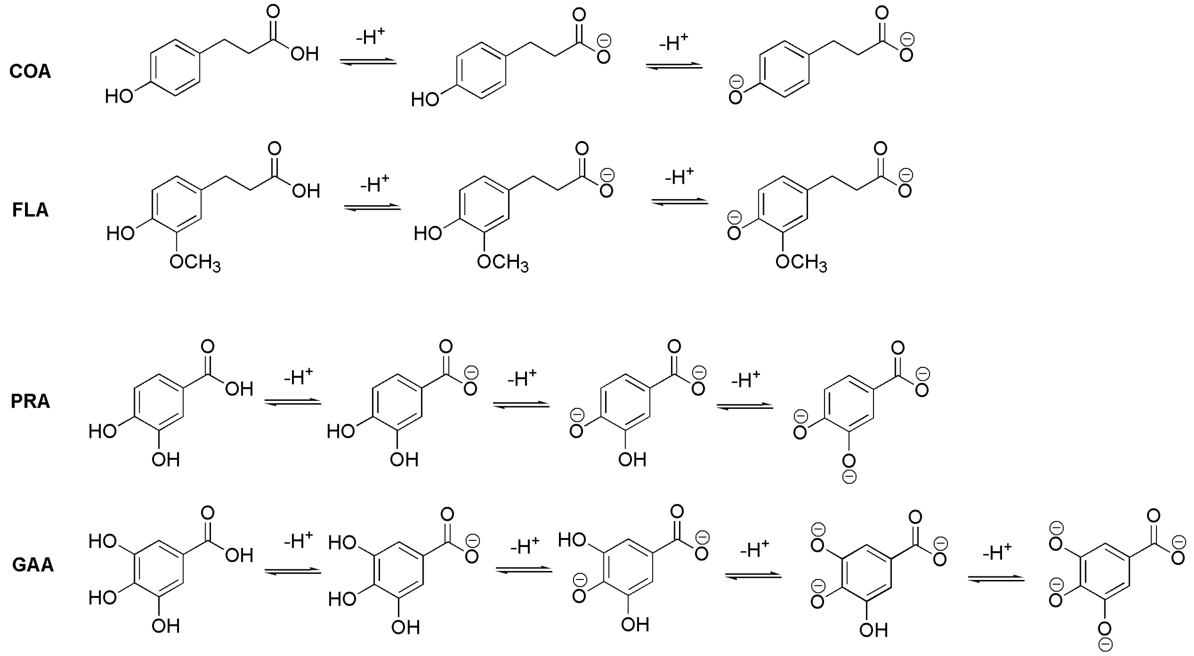

Figure S2. Deprotonation routes of PhAs

Table S1. Chelation routes (ChR), chelation sites (ChS), conditional Gibbs free energies of reaction (ΔG', at pH=7.4, in kcal/mol), and Maxwell-Boltzmann distribution (%MB) for the chelation pathways of PhAs.

Complex	ChS	ChR	ΔG	%MB	Structure	3D View
H_nCOA(**1**)-C1	CO	I	3.06	0	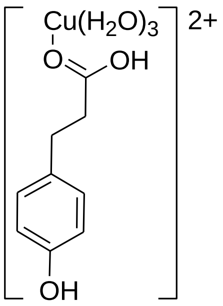	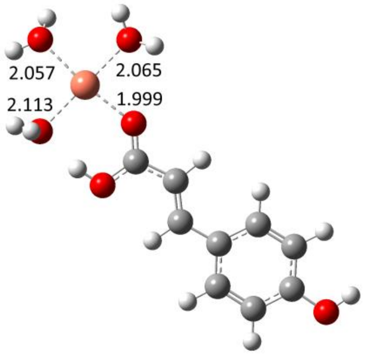
H_nCOA(**1**)-C2	CO	II	-11.19	0	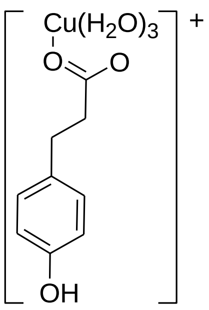	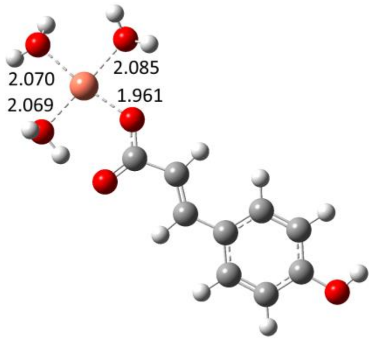
H_nCOA(**2**)-C3	COO	II	-15.51	0	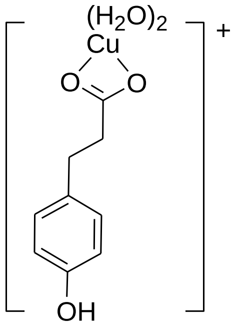	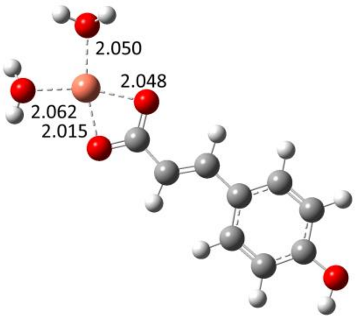

H_nCOA(**1**)-C4	OH	I	7.13	0	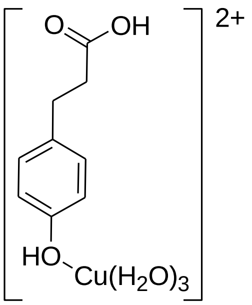	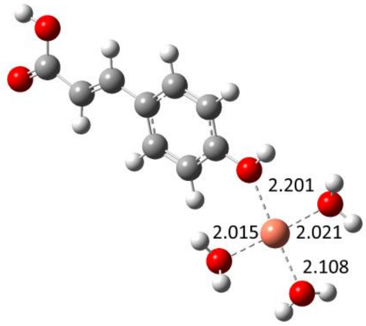
H_nCOA(**1**)-C5	OH	II	-6.34	0	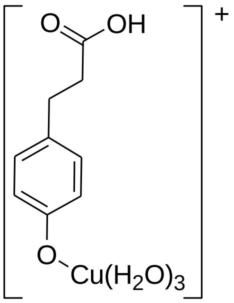	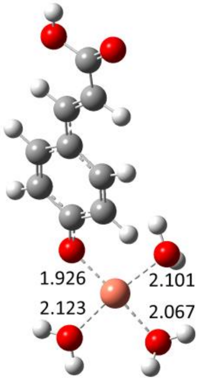
$H_{n-1}COA^{-}$(**1**)-C1	CO	III	-10.80	0	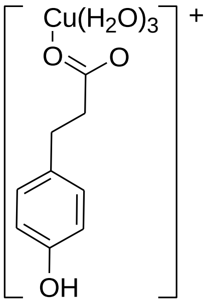	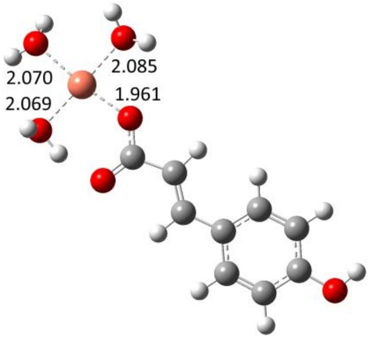
$H_{n-1}COA^{-}$(**2**)-C2	COO	III	-15.11	0	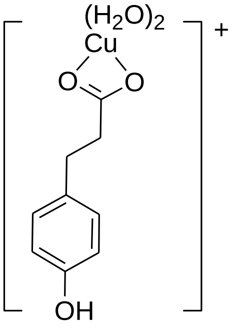	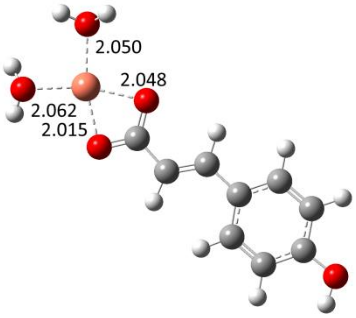

$H_{n-1}COA^{-}$ (**1**)-C3	OH	III	5.55	0	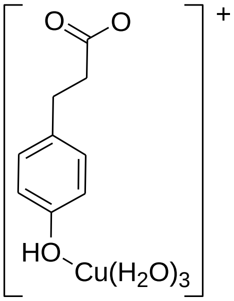	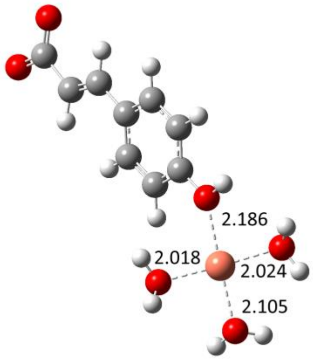
$H_{n-1}COA^{-}$ (**1**)-C4	OH	IV	-5.93	0	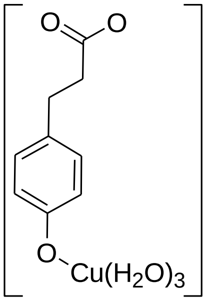	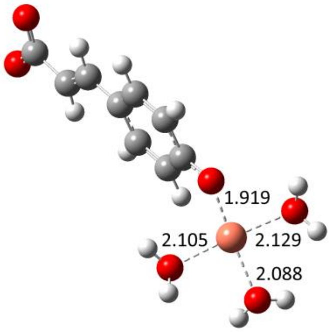
$H_{n-2}COA^{2-}$ (**1**)-C1	CO	V	-12.99	0	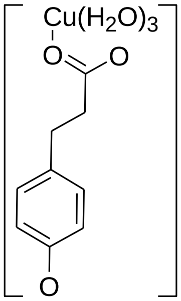	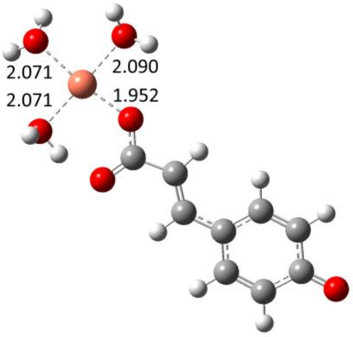
$H_{n-2}COA^{2-}$ (**2**)-C2	COO	V	-22.87	~100	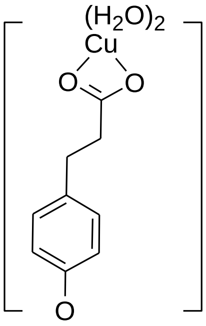	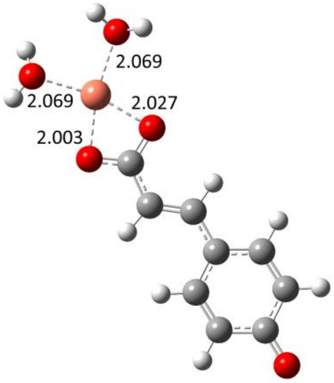

$H_{n-2}COA^{2-}$ (**1**)-C3	OH	V	-12.77	0	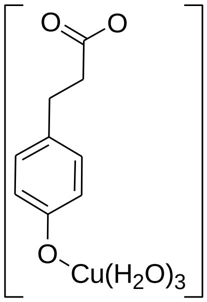	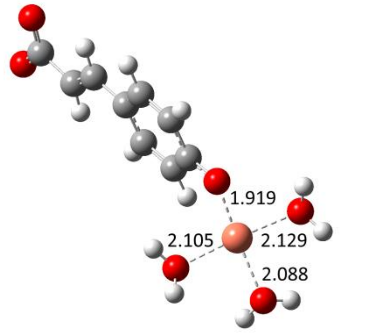
H_nFLA(**1**)-C1	CO	I	2.51	0	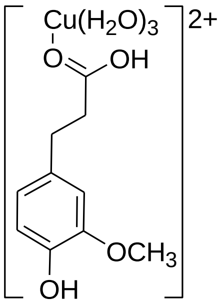	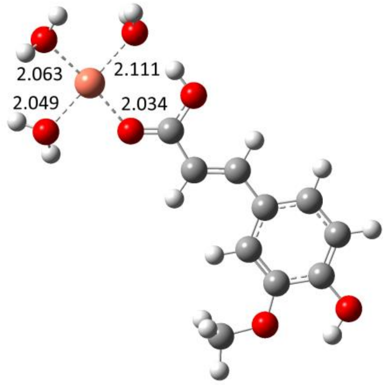
H_nFLA (**1**)-C2	CO	II	-10.99	0	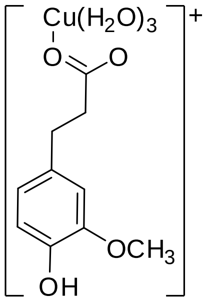	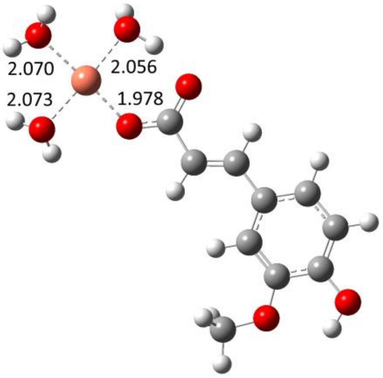
H_nFLA (**2**)-C3	COO	II	-15.43	0.14	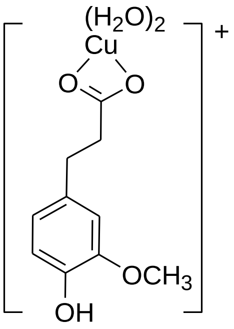	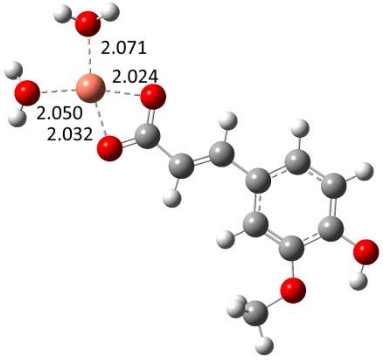

H_nFLA (**1**)-C4	OH	I	7.50	0	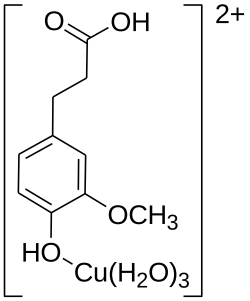	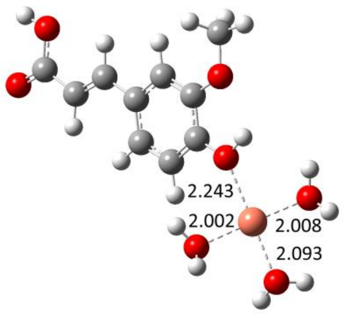
H_nFLA (**1**)-C5	OH	II	-6.35	0	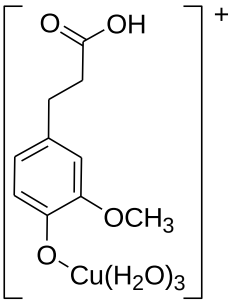	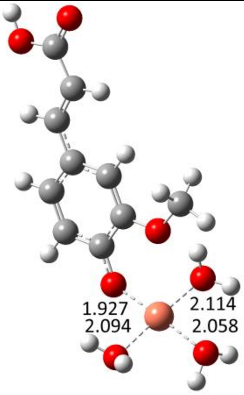
H_nFLA (**1**)-C6	OCH_3	I	7.05	0	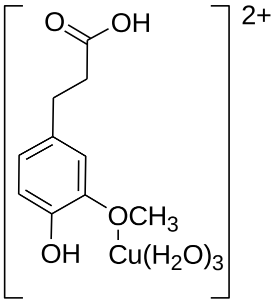	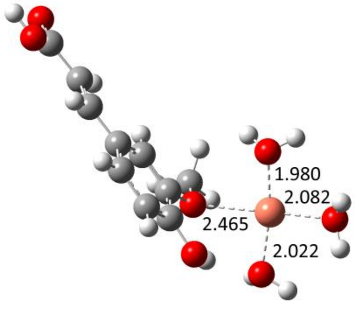
H_nFLA (**2**)-C7	OH, OCH_3	I	4.40	0	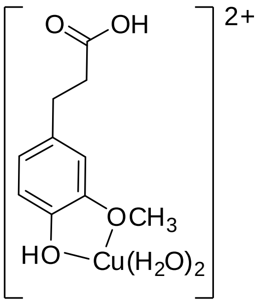	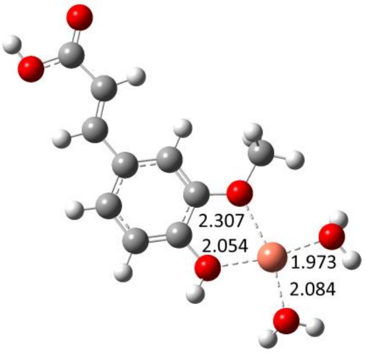

H_nFLA (**2**)-C8	OH, OCH_3	II	-13.23	0	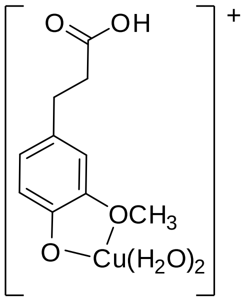	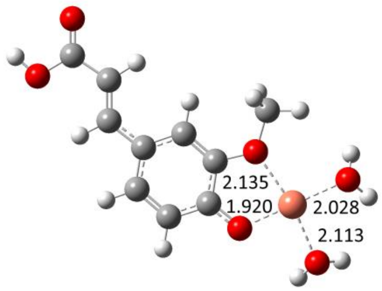
$H_{n-1}FLA^-$ (**1**)-C1	CO	III	-9.96	0	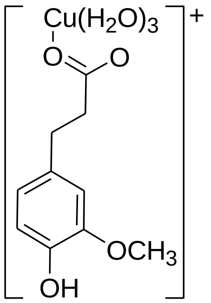	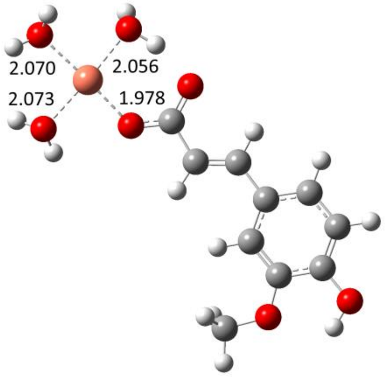
$H_{n-1}FLA^-$ (**2**)-C2	COO	III	-14.40	0.03	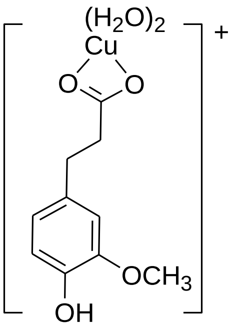	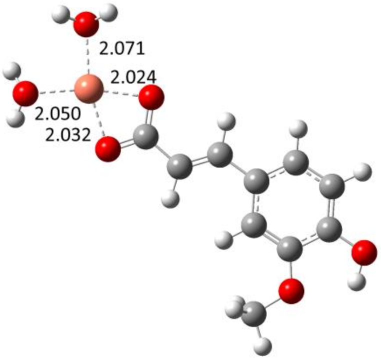
$H_{n-1}FLA^-$ (**1**)-C3	OH	III	6.28	0	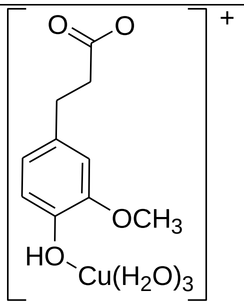	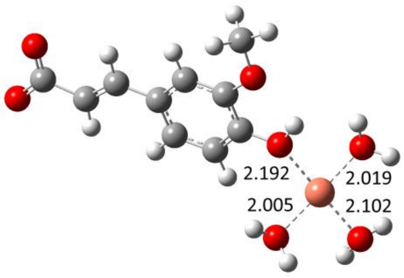

$H_{n-1}FLA^{-}$ (**1**)-C4	OH	IV	-5.92	0	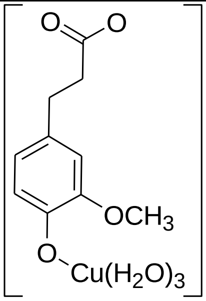	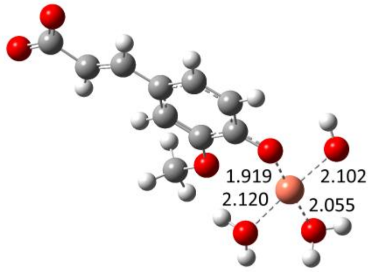
$H_{n-1}FLA^{-}$ (**1**)-C5	OCH_3	III	6.22	0	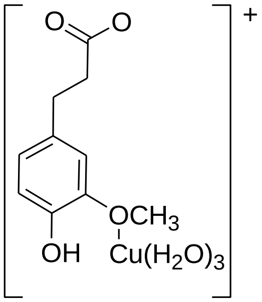	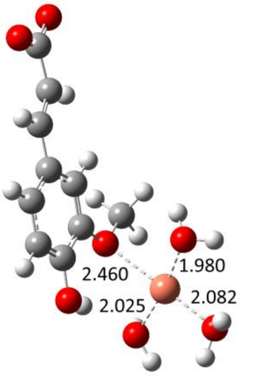
$H_{n-1}FLA^{-}$ (**2**)-C6	OH, OCH_3	III	3.13	0	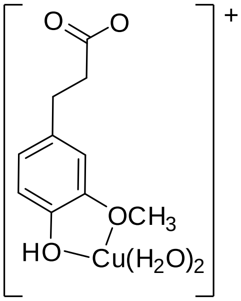	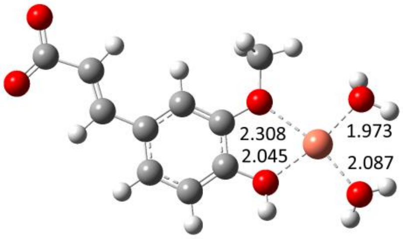
$H_{n-1}FLA^{-}$ (**2**)-C7	OH, OCH_3	IV	-12.4	0	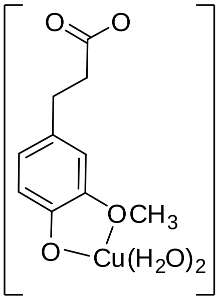	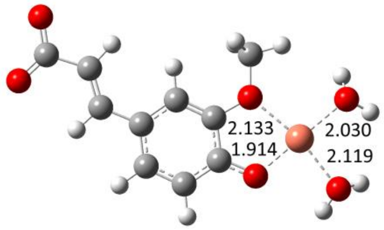

$H_{n-2}FLA^{2-}$ (**1**)-C1	CO	V	-12.08	0	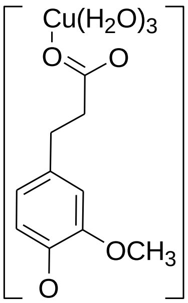	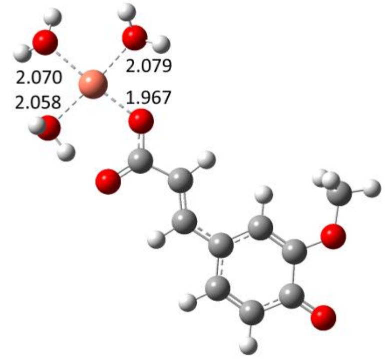
$H_{n-2}FLA^{2-}$ (**2**)-C2	COO	V	-18.30	18.21	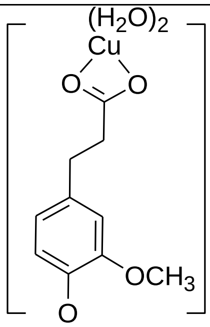	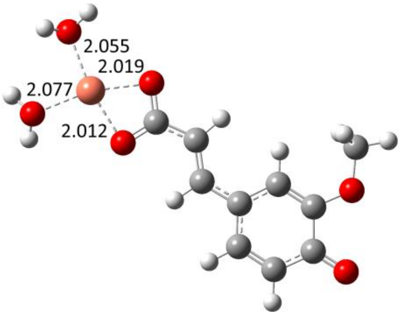
$H_{n-2}FLA^{2-}$ (**1**)-C3	OH	V	-12.67	0	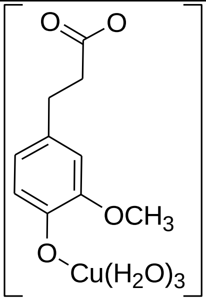	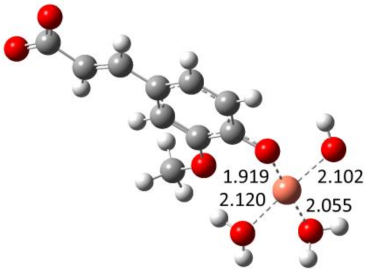
$H_{n-2}FLA^{2-}$ (**1**)-C4	OCH_3	V	-3.50	0	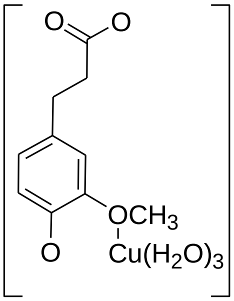	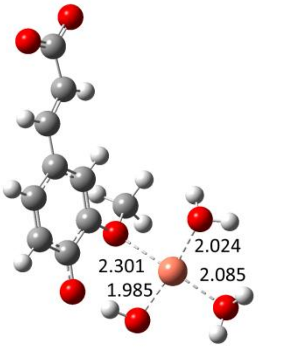

$H_{n-2}FLA^{2-}$ (**2**)-C5	OH, OCH_3	V	-19.19	81.61	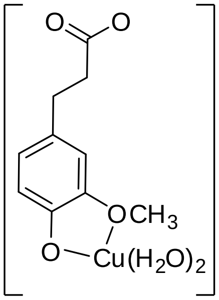	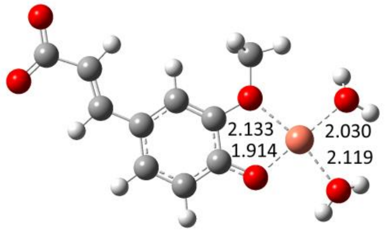
H_nPRA(**1**)-C1	CO	I	2.05	0	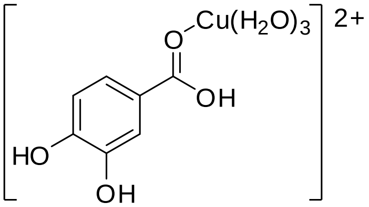	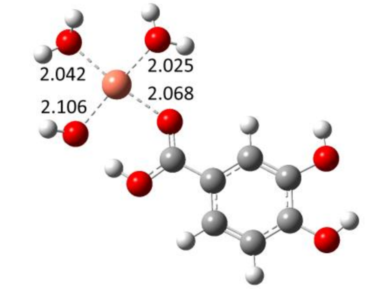
H_nPRA (**1**)-C2	CO	II	-13.54	0	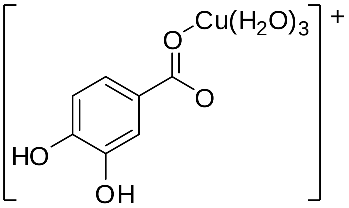	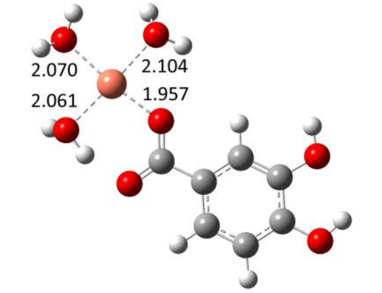
H_nPRA (**2**)-C3	COO	II	-16.57	0	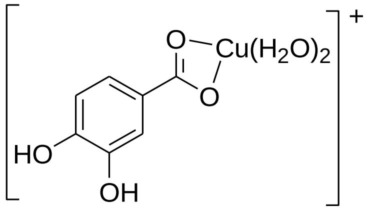	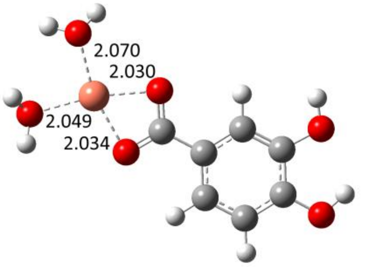
H_nPRA (**1**)-C4	OH (3)	I	5.51	0	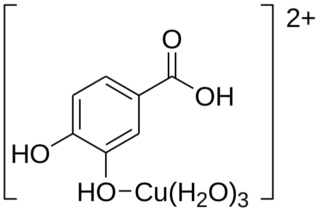	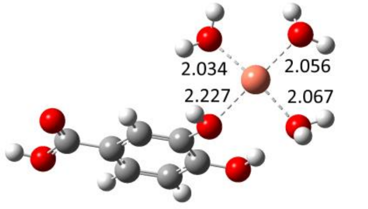

H_nPRA (**1**)-C5	OH (3)	II	-9.26	0	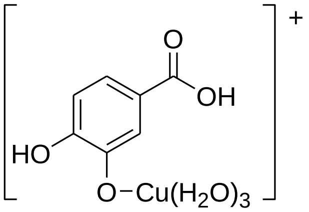	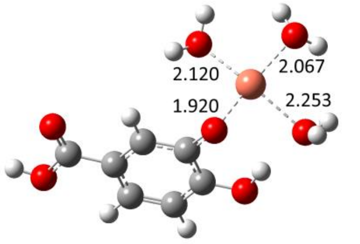
H_nPRA (**1**)-C6	OH (4)	I	5.74	0	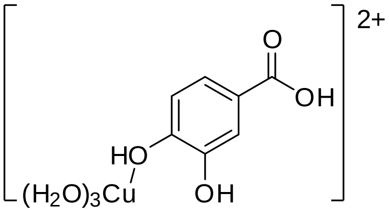	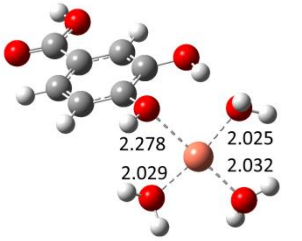
H_nPRA (**1**)-C7	OH (4)	II	-9.33	0	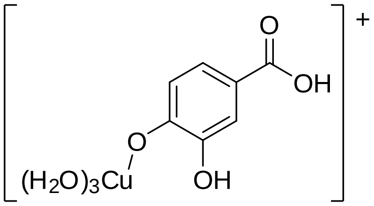	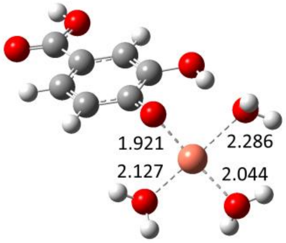
H_nPRA (**2**)-C8	OH (3), OH (4)	I	3.55	0	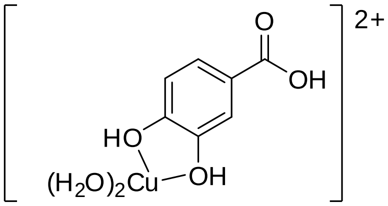	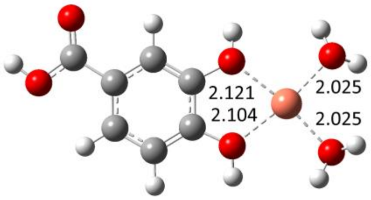
H_nPRA (**2**)-C9	OH (3), OH (4)	II	-13.04	0	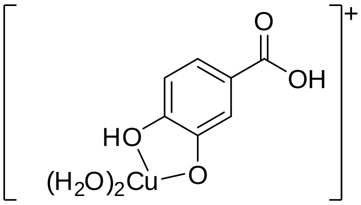	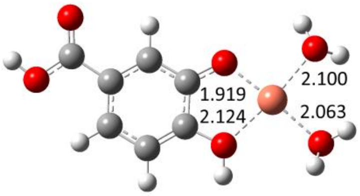
H_nPRA (**2**)-C10	OH (3), OH (4)	II	-13.21	0	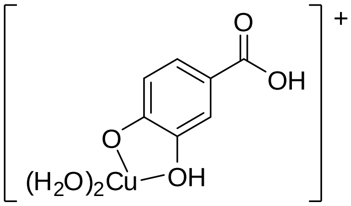	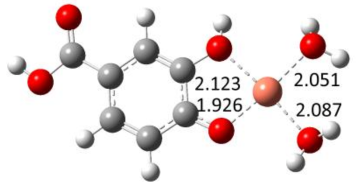

$H_{n-1}PRA^{-}$ (**1**)-C1	CO	III	-11.19	0	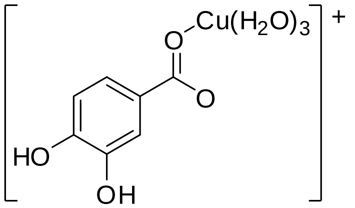	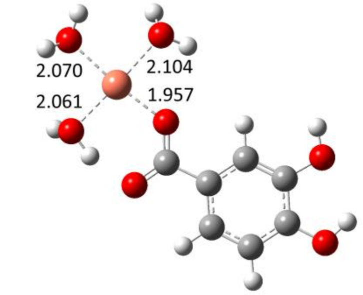
$H_{n-1}PRA^{-}$ (**2**)-C2	COO	III	-14.22	0	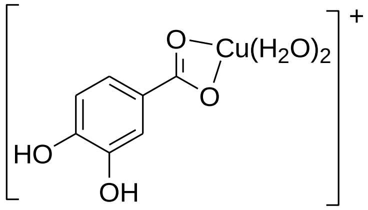	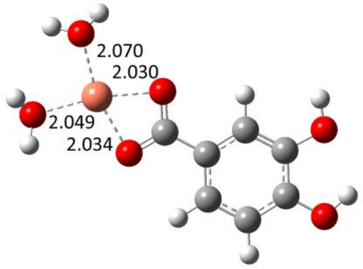
$H_{n-1}PRA^{-}$ (**1**)-C3	OH (3)	III	4.30	0	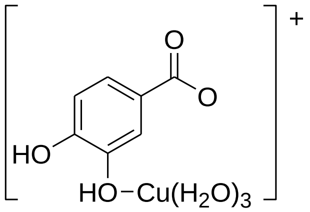	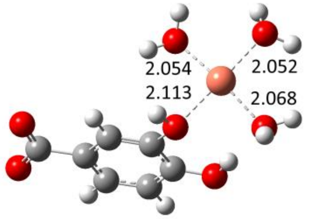
$H_{n-1}PRA^{-}$ (**1**)-C4	OH (3)	IV	-8.77	0	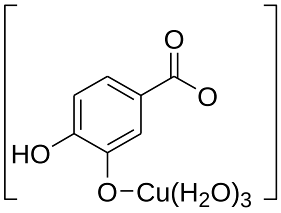	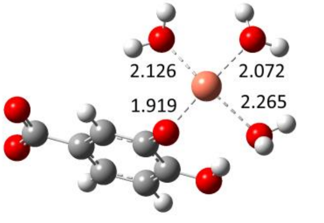
$H_{n-1}PRA^{-}$ (**1**)-C5	OH (4)	III	6.61	0	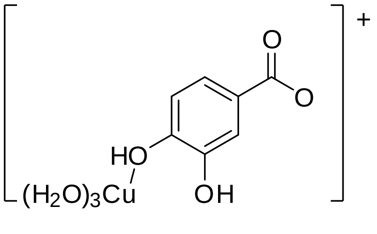	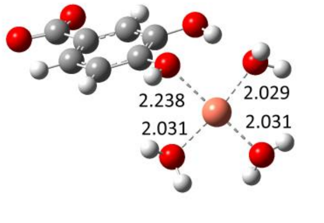

$H_{n-1}PRA^{-}$ (**1**)-C6	OH (4)	IV	-9.58	0	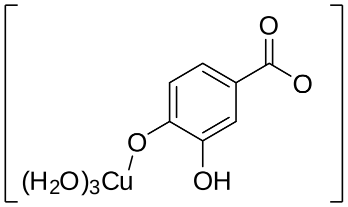	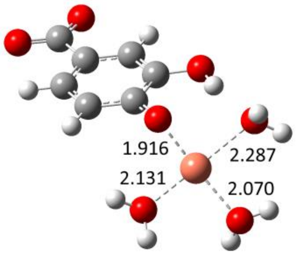
$H_{n-1}PRA^{-}$ (**2**)-C7	OH (3), OH (4)	III	1.79	0	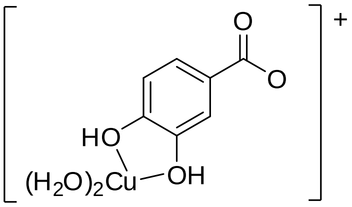	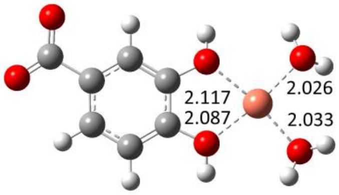
$H_{n-1}PRA^{-}$ (**2**)-C8	OH (3), OH (4)	IV	-12.15	0	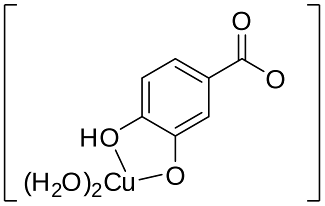	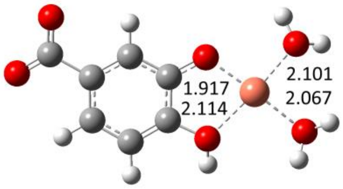
$H_{n-1}PRA^{-}$ (**2**)-C9	OH (3), OH (4)	IV	-12.58	0	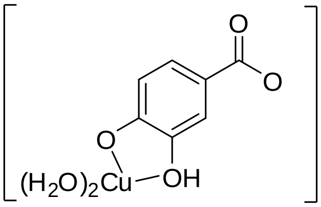	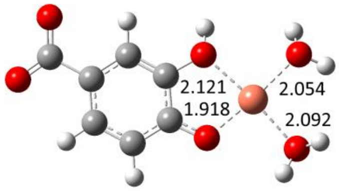
$H_{n-2}PRA^{2-}$ (**1**)-C1	CO	V	-10.97	0	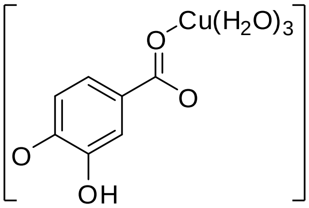	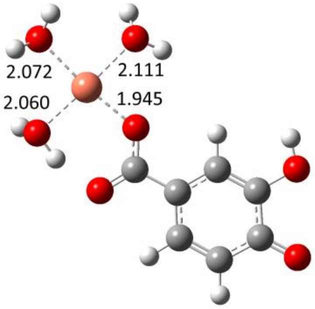

$H_{n-2}PRA^{2-}$ (**2**)-C2	COO	V	-16.23	0	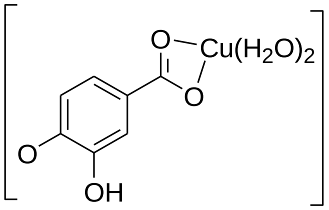	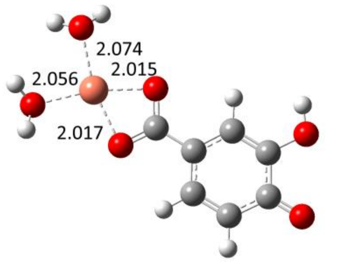
$H_{n-2}PRA^{2-}$ (**1**)-C3	OH (3)	V	-3.17	0	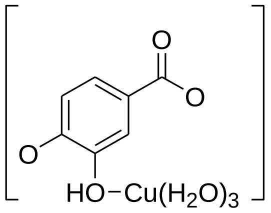	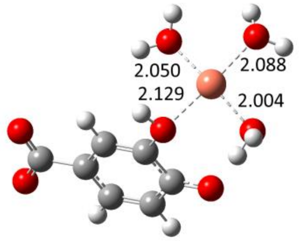
$H_{n-2}PRA^{2-}$ (**1**)-C4	OH (3)	VI	-12.02	0	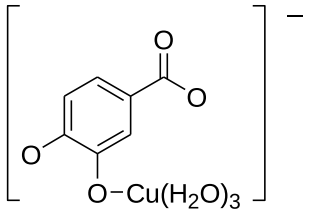	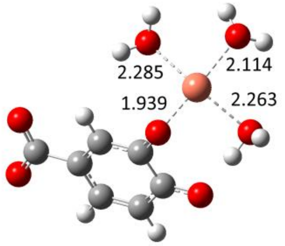
$H_{n-2}PRA^{2-}$ (**1**)-C5	OH (4)	V	-14.42	0	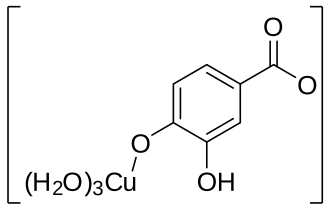	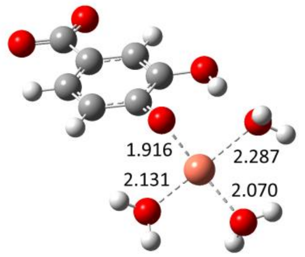
$H_{n-2}PRA^{2-}$ (**2**)-C6	OH (3), OH (4)	V	-17.42	0.01	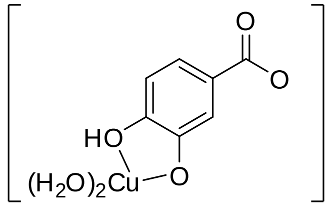	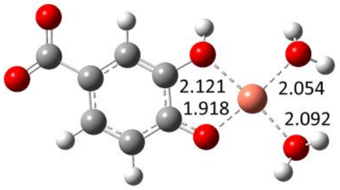
$H_{n-2}PRA^{2-}$ (**2**)-C7	OH (3), OH (4)	VI	-22.83	99.99	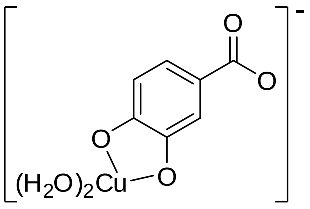	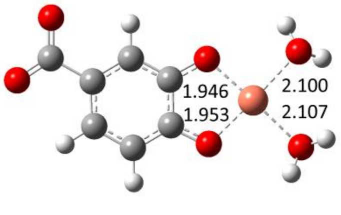

H_nGAA(**1**)-C1	CO	I	2.40	0	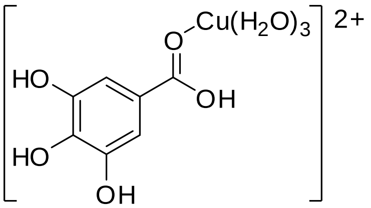	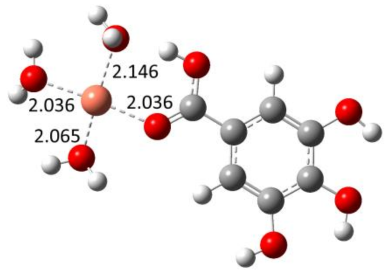
H_nGAA (**1**)-C2	CO	II	-13.70	0	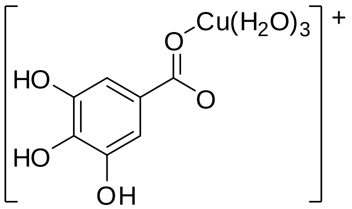	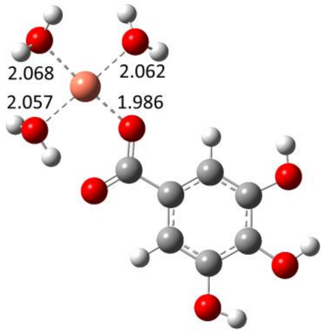
H_nGAA (**2**)-C3	COO	II	-16.65	0.08	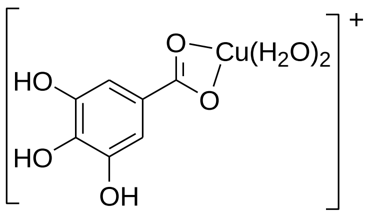	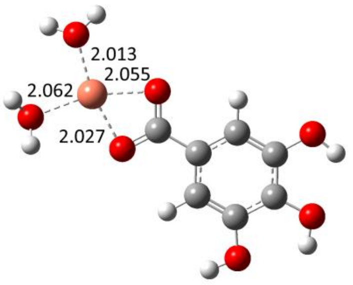
H_nGAA (**1**)-C4	OH (3)	I	6.93	0	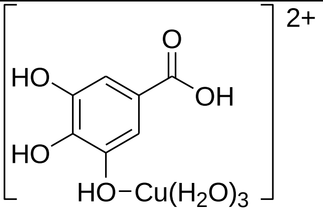	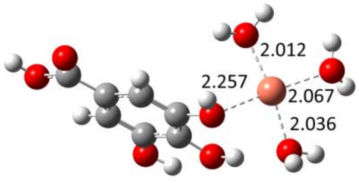
H_nGAA (**1**)-C5	OH (3)	II	-8.26	0	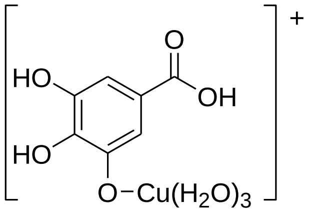	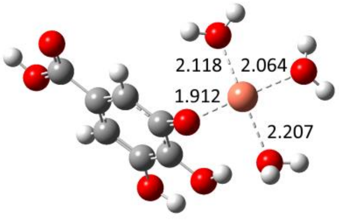

H_nGAA (**1**)-C6	OH (4)	I	5.55	0	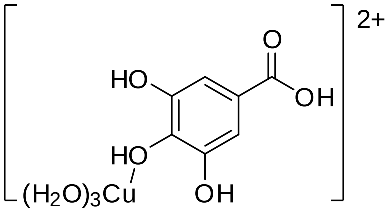	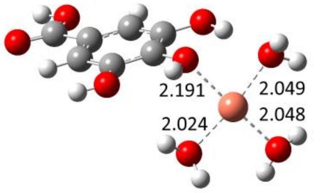
H_nGAA (**1**)-C7	OH (4)	II	-11.01	0	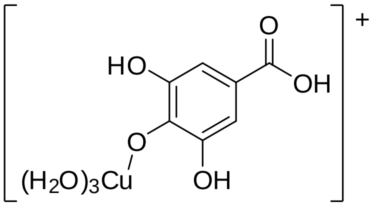	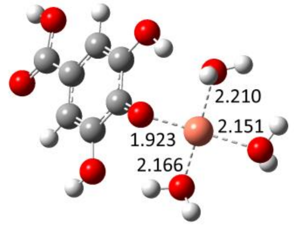
H_nGAA (**2**)-C8	OH (3),<br>OH (4)	I	3.61	0	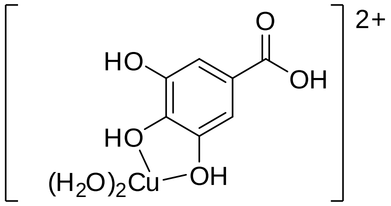	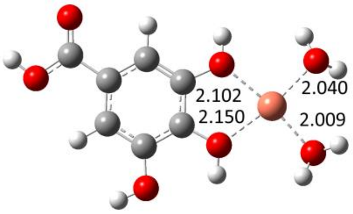
H_nGAA (**2**)-C9	OH (3),<br>OH (4)	II	-13.00	0	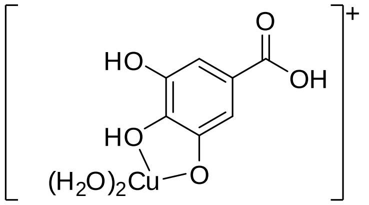	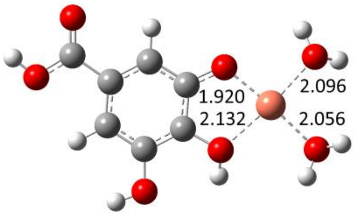
H_nGAA N(**2**)-C10	OH (3),<br>OH (4)	II	-12.77	0	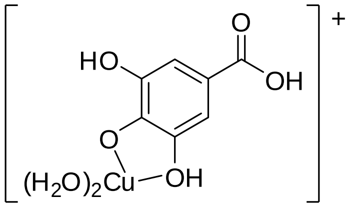	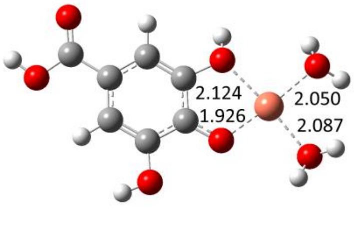

$H_{n-1}GAA^-$ (**1**)-C1	CO	III	-10.93	0	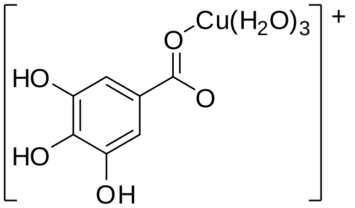	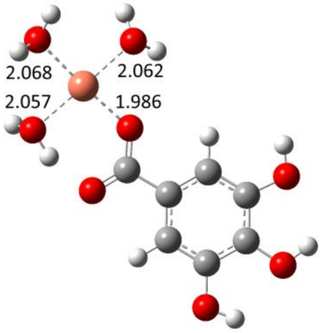
$H_{n-1}GAA^-$ (**2**)-C2	COO	III	-13.87	0	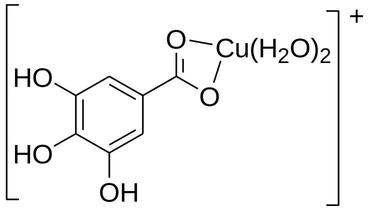	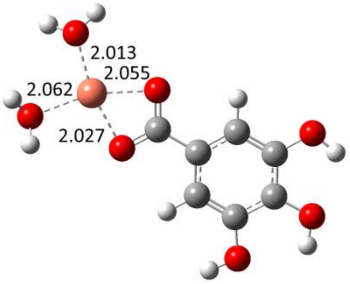
$H_{n-1}GAA^-$ (**1**)-C3	OH (3)	III	5.98	0	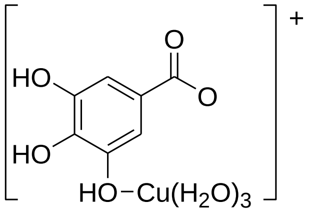	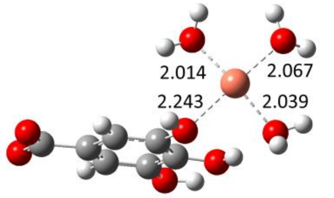
$H_{n-1}GAA^-$ (**1**)-C4	OH (3)	IV	-7.94	0	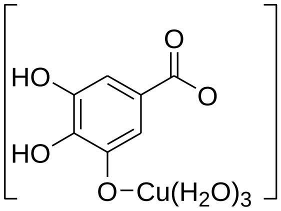	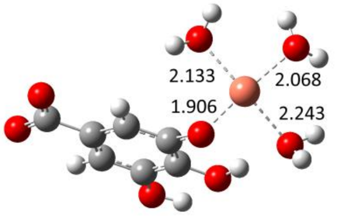
$H_{n-1}GAA^-$ (**1**)-C5	OH (4)	III	4.80	0	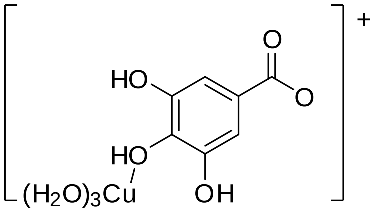	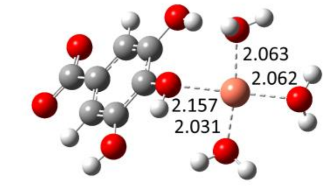

$H_{n-1}GAA^{-}$ (**1**)-C6	OH (4)	IV	-11.42	0	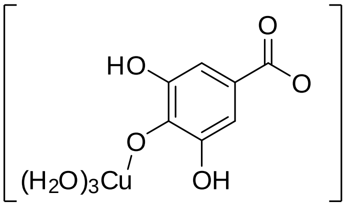	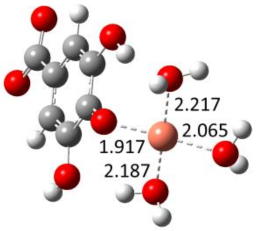
$H_{n-1}GAA^{-}$ (**2**)-C7	OH (3), OH (4)	III	2.04	0	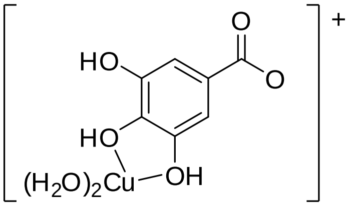	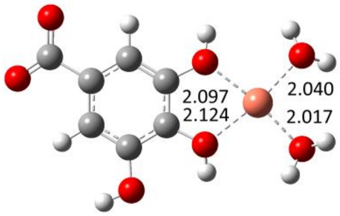
$H_{n-1}GAA^{-}$ (**2**)-C8	OH (3), OH (4)	IV	-11.97	0	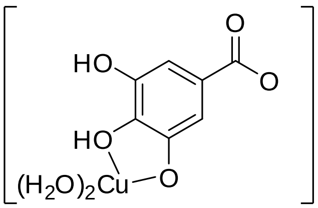	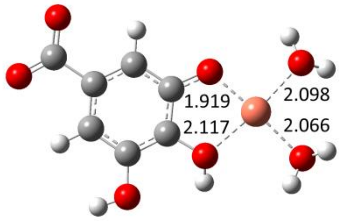
$H_{n-1}GAA^{-}$ (**2**)-C9	OH (3), OH (4)	IV	-11.57	0	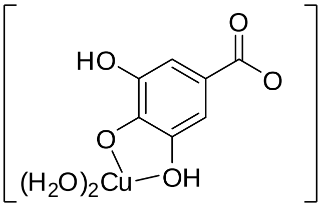	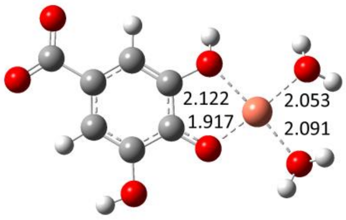
$H_{n-2}GAA^{2-}$ (**1**)-C1	CO	V	-13.82	0	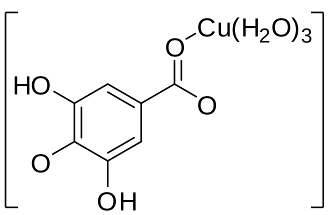	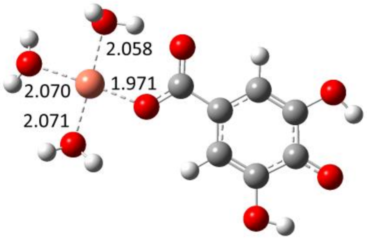

$H_{n-2}GAA^{2-}$ (**2**)-C2	COO	V	-18.48	1.86	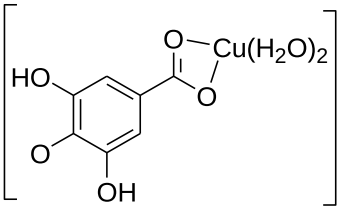	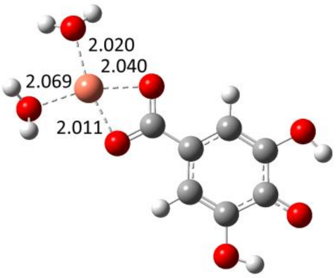
$H_{n-2}GAA^{2-}$ (**1**)-C3	OH (3)	V	-2.85	0	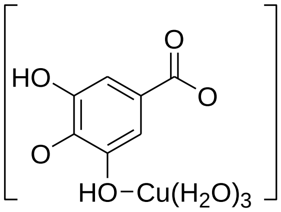	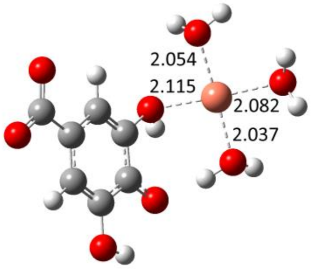
$H_{n-2}GAA^{2-}$ (**1**)-C4	OH (3)	VI	-11.54	0	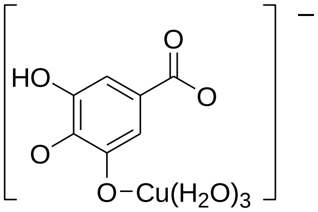	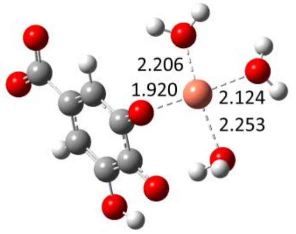
$H_{n-2}GAA^{2-}$ (**1**)-C5	OH (4)	V	-14.30	0	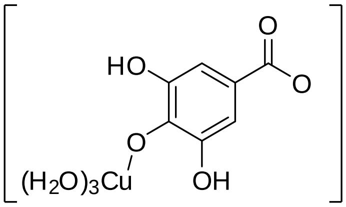	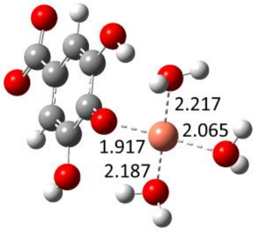
$H_{n-2}GAA^{2-}$ (**2**)-C6	OH (3), OH (4)	V	-14.45	0	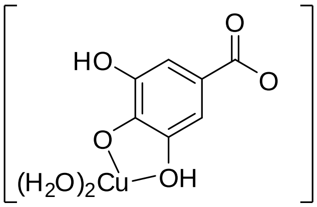	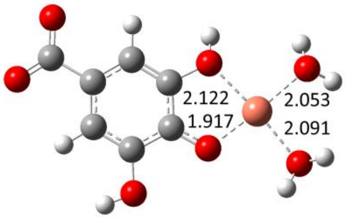

$H_{n-2}GAA^{2-}$ (**2**)-C7	OH (3), OH (4)	VI	-20.83	98.05	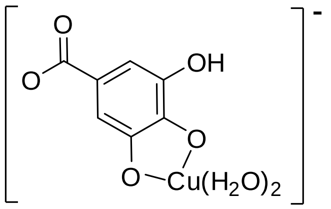	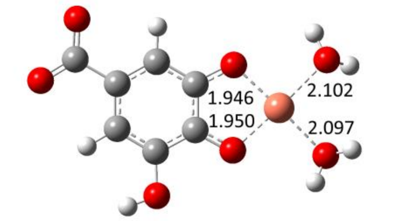

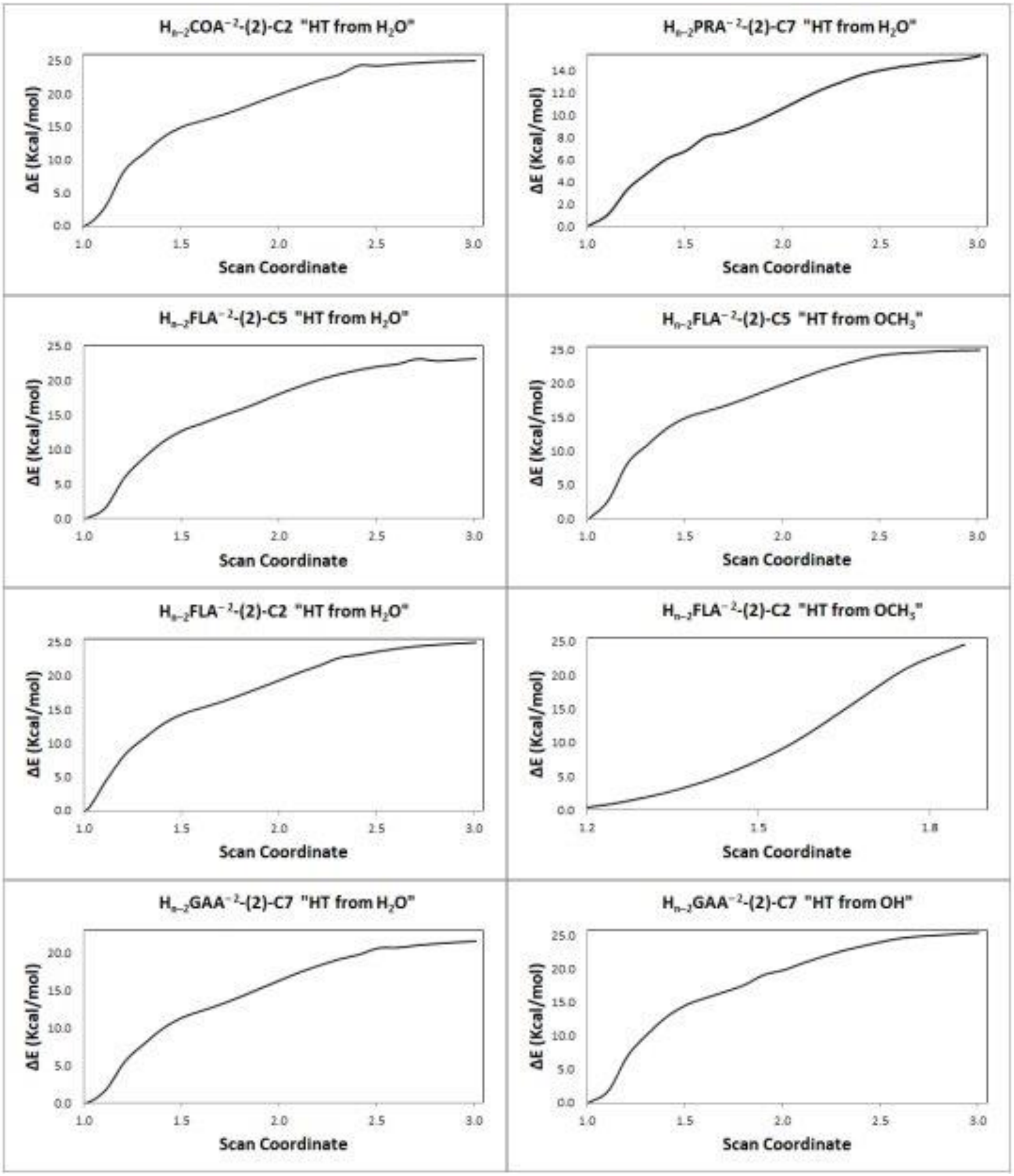

Figure S3. Energy scan for the *f*-HAT reactions between •OH and PhAs-Cu(II) at 298.15 K. Solvent=water.

Table S2. Gibbs free energy of reaction (ΔG, kcal/mol) and Gibbs free energy of activation (ΔG≠, kcal/mol) for the direct RAF mechanism between PhAs-Cu(II) and •OH. Solvent=water.

**PhAs-Cu(II)**		**ΔG**	**ΔG≠**
$H_{n-2}COA^{2-}$ (2)-C2	RAF-1	-8.10	-1.74
$H_{n-2}COA^{2-}$ (2)-C2	RAF-2	-12.92	-1.38
$H_{n-2}COA^{2-}$ (2)-C2	RAF-3	-17.08	-6.81
$H_{n-2}COA^{2-}$ (2)-C2	RAF-4	-13.63	-6.28
$H_{n-2}FLA^{2-}$ (2)-C5	RAF-1	-10.16	-2.48
$H_{n-2}FLA^{2-}$ (2)-C5	RAF-2	NF	--
$H_{n-2}FLA^{2-}$ (2)-C5	RAF-3	-18.40	-4.78
$H_{n-2}FLA^{2-}$ (2)-C5	RAF-4	-20.79	-8.05
$H_{n-2}FLA^{2-}$ (2)-C5	RAF-5	-15.21	-4.43
$H_{n-2}FLA^{2-}$ (2)-C5	RAF-6	-17.52	-6.38
$H_{n-2}FLA^{2-}$ (2)-C2	RAF-1	-8.11	-3.01
$H_{n-2}FLA^{2-}$ (2)-C2	RAF-2	-15.39	-3.32
$H_{n-2}FLA^{2-}$ (2)-C2	RAF-3	-20.84	-7.25
$H_{n-2}FLA^{2-}$ (2)-C2	RAF-4	-15.78	-9.98
$H_{n-2}FLA^{2-}$ (2)-C2	RAF-5	-14.96	-6.56
$H_{n-2}FLA^{2-}$ (2)-C2	RAF-6	-16.08	-5.88
$H_{n-2}PRA^{2-}$ (2)-C7	RAF-1	-15.32	-6.22
$H_{n-2}PRA^{2-}$ (2)-C7	RAF-2	-17.03	-8.33
$H_{n-2}PRA^{2-}$ (2)-C7	RAF-3	-17.50	-12.01
$H_{n-2}PRA^{2-}$ (2)-C7	RAF-4	-18.97	-12.62
$H_{n-2}PRA^{2-}$ (2)-C7	RAF-5	-14.90	-7.18
$H_{n-2}PRA^{2-}$ (2)-C7	RAF-6	-19.63	-10.62
$H_{n-2}GAA^{2-}$ (2)-C7	RAF-1	-14.88	-6.36
$H_{n-2}GAA^{2-}$ (2)-C7	RAF-2	-18.24	-7.63
$H_{n-2}GAA^{2-}$ (2)-C7	RAF-3	-15.82	-10.74
$H_{n-2}GAA^{2-}$ (2)-C7	RAF-4	-19.96	-13.07
$H_{n-2}GAA^{2-}$ (2)-C7	RAF-5	-19.19	-9.09
$H_{n-2}GAA^{2-}$ (2)-C7	RAF-6	-17.74	-9.46

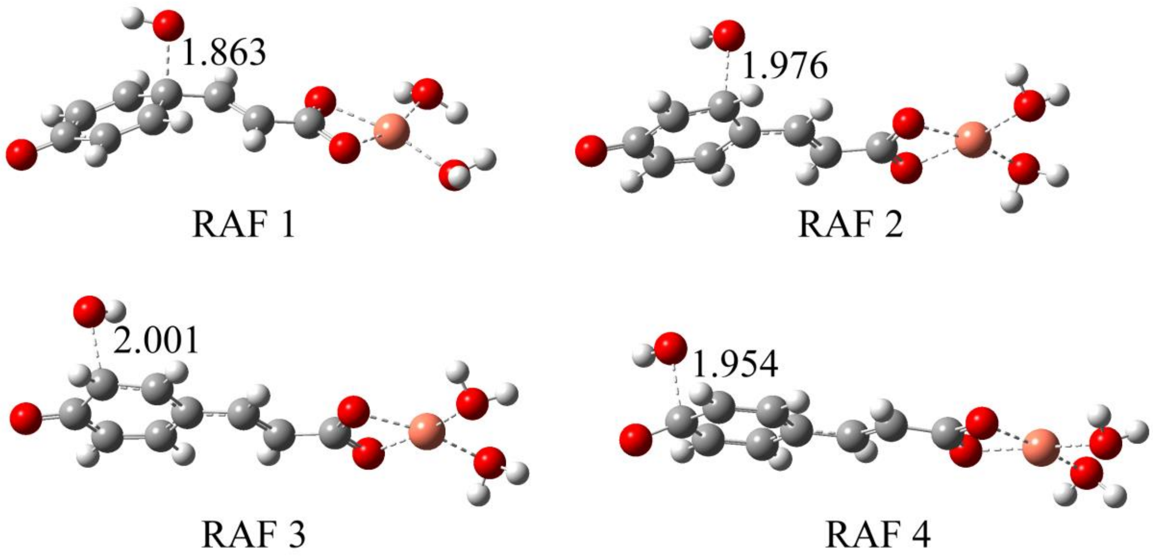

Figure S4. Transition states structures of exergonic RAF reactions between $^{\bullet}OH$ and $H_{n-2}COA^{2-}$-(2)-C2 at 298.15 K. Solvent=water.

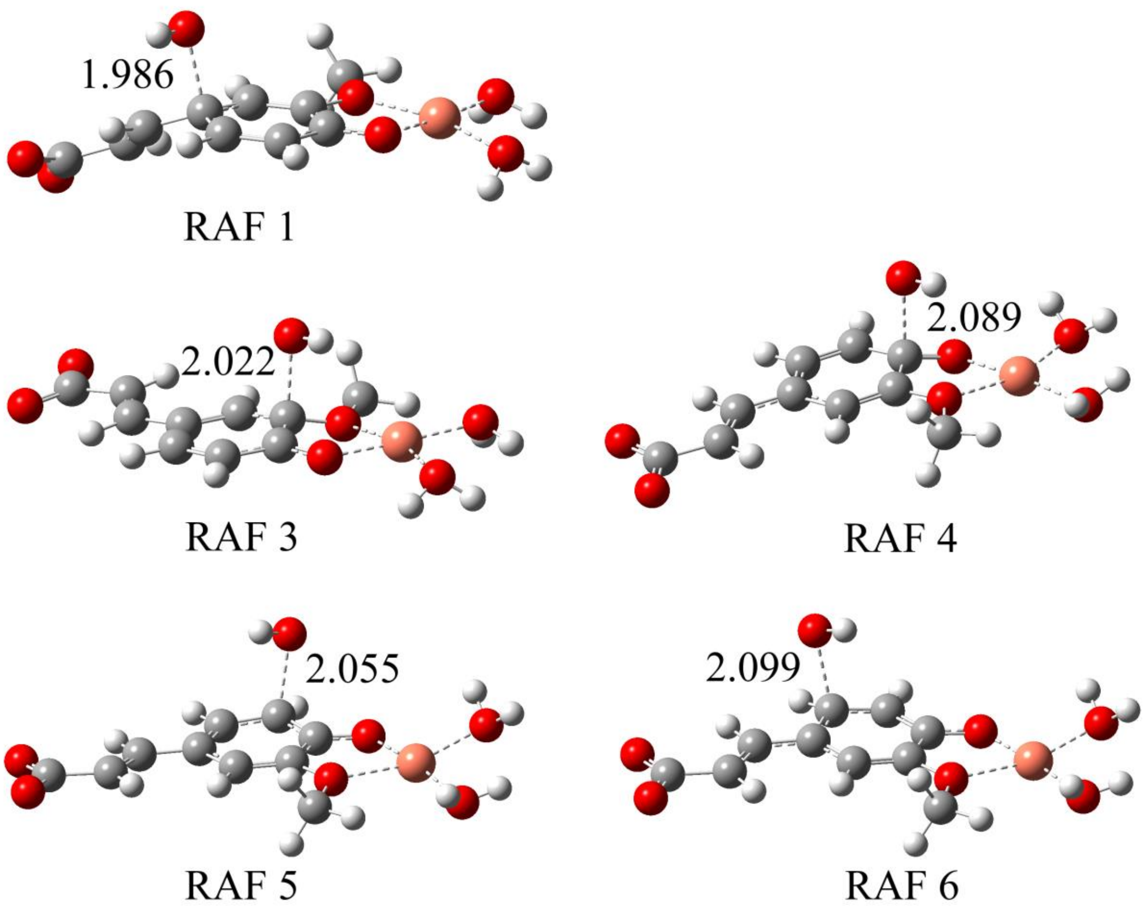

Figure S5. Transition states structures of exergonic RAF reactions between $^{\bullet}OH$ and $H_{n-2}FLA^{2-}$-(2)-C5 complexes at 298.15 K. Solvent=water.

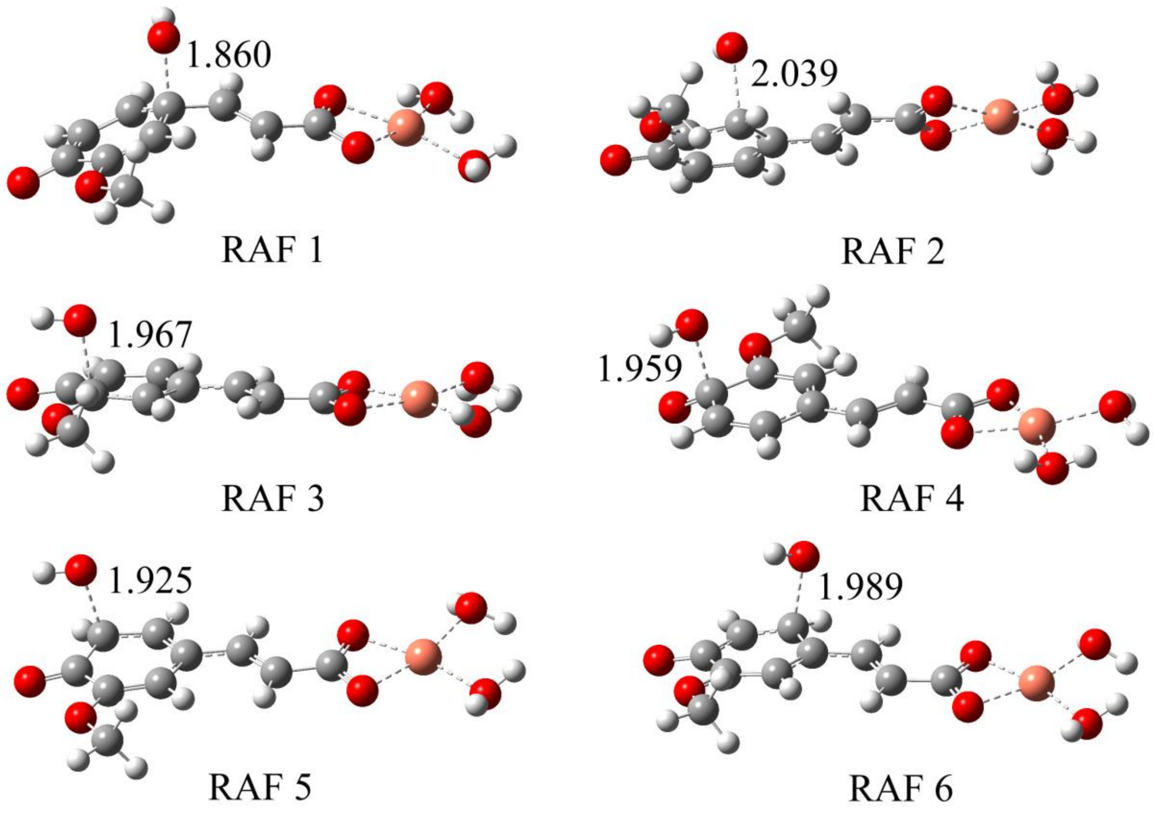

Figure S6. Transition states structures of exergonic RAF reactions between $^{\bullet}$OH and $H_{n-2}FLA^{2-}$-(2)-C2 complexes at 298.15 K. Solvent=water.

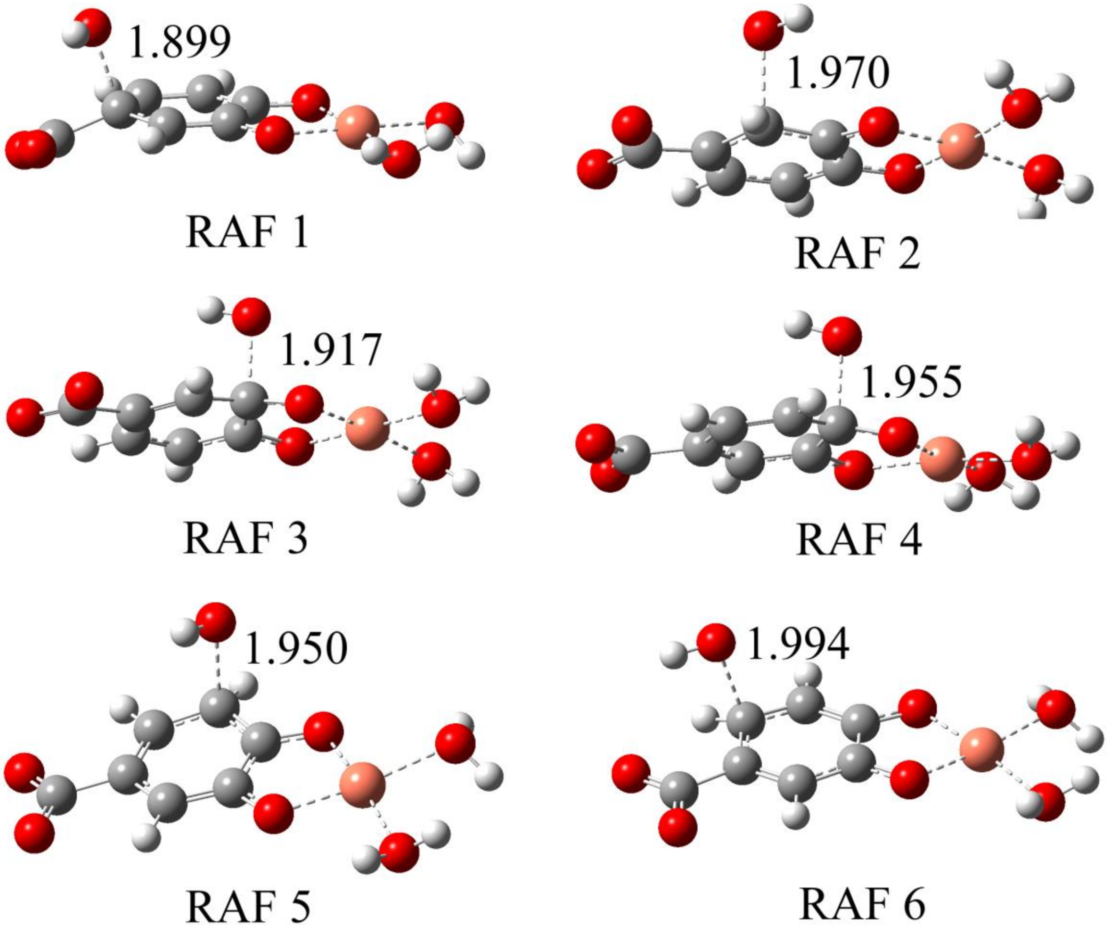

Figure S7. Transition states structures of exergonic RAF reactions between •OH and $H_{n-2}PRA^{2-}$-(2)-C7 complexes at 298.15 K. Solvent=water.

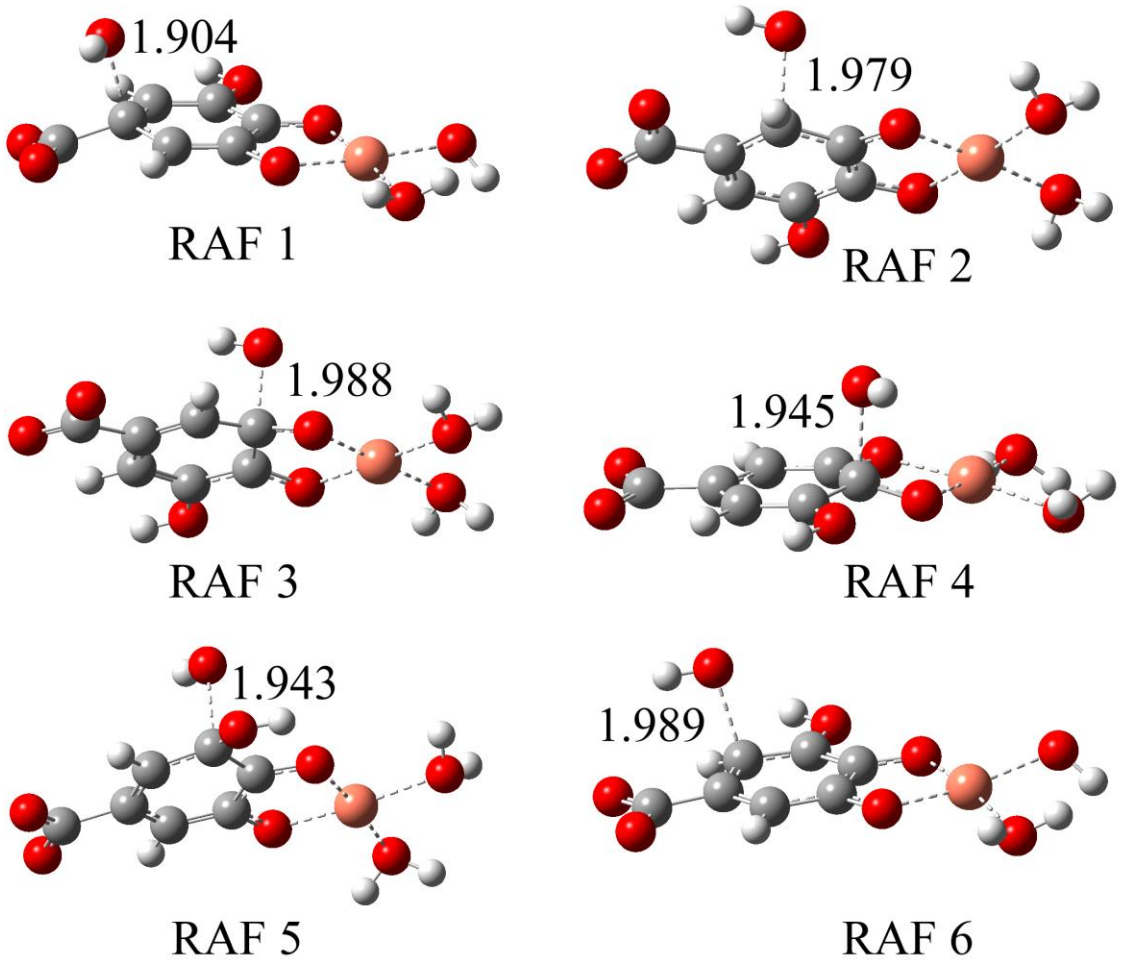

Figure S8. Transition states structures of exergonic RAF reactions between $^{\bullet}OH$ and $H_{n-2}GAA^{2-}$-(2)-C7 complexes at 298.15 K. Solvent=water.
